# A systematic review and meta-analysis of the effects of errorless motor learning on movement outcomes: a lifespan and impairment perspective

**DOI:** 10.3389/fpsyg.2026.1722743

**Published:** 2026-03-24

**Authors:** Yuen Ting Wong, Hoi Kwan Yuen, Timothy T. T. Yam, William W. N. Tsang, Liis Uiga, Catherine M. Capio

**Affiliations:** 1 Department of Physiotherapy, School of Nursing and Health Sciences, Hong Kong Metropolitan University, Hong Kong, Hong Kong SAR, China; 2 Department of Sport and Exercise Sciences, Manchester Metropolitan University, Manchester, United Kingdom

**Keywords:** children, errorless learning, impairment, motor learning, older adults

## Abstract

**Background:**

When practice is structured such that learners experience considerable success, movement skills are learned effectively. Errorless motor learning minimises errors during practice by constraining the environment to promote success in the early stage of practice. This systematic review aims to synthesise the evidence on the effects of errorless motor learning on performance outcomes (OSF registration: 10.17605/OSF.IO/SPHR6).

**Method:**

A literature search of nine electronic databases (MEDLINE, Embase, Cochrane Library, PubMed, CINAHL Plus, PyscINFO, Scopus, Web of Science, and PEDro) and grey literature (Google Scholar and ProQuest Open Dissertation) identified experimental and quasi-experimental studies that examined the effect of errorless motor learning on movement task performance. Effect sizes were calculated (Hedges’ g) and heterogeneity was assessed using the Q-statistic and *I*^2^ index. Risk of bias was assessed using ROBINS-I or RoB2 and certainty of evidence using GRADE.

**Results:**

Out of the 8,514 articles from the search, 29 (31 experiments), including a total of 1,509 participants, were eligible. For the full sample analysis of movement performance, errorless motor learning did not demonstrate a significant overall effect compared to errorful learning (*g* = 0.0506, *p* = 0.223), nor were significant effects found among children (*p* = 0.068) or young adults (*p* = 0.925). However, a significant large effect was observed among those with impairments (*g* = 0.804; *p* < 0.001). For movement accuracy, a significant moderate overall effect was found (*g* = 0.854; *p* = 0.023), among children (*g* = 0.657; *p* = 0.007) and learners with impairments (*g* = 0.873; *p* = 0.004), but not among young adults (*p* = 0.303) or learners without impairments (*p* = 0.117). Risk of bias varied from some concerns/moderate risk of bias to serious/high risk of bias. GRADE ratings ranged from very low to low.

**Conclusion:**

Errorless motor learning did not demonstrate a consistent overall advantage over errorful learning for movement outcomes. Significant benefits are apparent in specific subgroups – children and individuals with impairments. The overall strength of the evidence is limited by moderate to high risk of bias, substantial heterogeneity, and low certainty across studies. Future research should employ more rigorous experimental designs to strengthen the evidence base and clarify conditions under which errorless learning is most effective.

**Systematic review registration:**

https://doi.org/10.17605/OSF.IO/MZXDG.

## Introduction

Motor learning consists of the processes that enable individuals to acquire, modify, and refine proficiency following practice and experiences, giving rise to relatively lasting changes in their ability to execute skilled behaviours (Schmidt and Lee, 2020). Dynamic learning processes, both cognitive and perceptual in nature, facilitate the coding of various motor programs, leading to improvements in the control, stability, and accuracy of movements necessary for different tasks (Mosby, 2013). Consequently, the way in which practice is structured plays a decisive role in motor learning efficiency. Research into how practice should be structured has shown that movement skills are learned effectively when individuals experience considerable success during practice (Babič et al., 2025; Leech et al., 2022; Welsby et al., 2024).

While making errors during practice has been traditionally viewed as beneficial for motor skill learning by facilitating exploration and error correction (Seidler et al., 2013a,b), other researchers argue that frequent practice errors may hinder the development of accurate motor programs. Specifically, errors may impose excessive cognitive demands, limiting the automaticity of movement control and thereby disrupting effective skill acquisition and retention (Maxwell et al., 2017). Errorless motor learning is a strategy that aims to minimise practice errors by constraining the environment (e.g., through gradual increase in task difficulty) to promote success in the early stage of practice (Kleynen et al., 2020; Maxwell et al., 2001). In this motor learning strategy, feedback are readily accessible to learners; however, they are believed to rely less on conscious adjustment of their movements and accumulate less declarative knowledge than are learners who experience more practice errors (Maxwell et al., 2001). By reducing cognitive load and reliance on working memory (Maxwell et al., 2001; Zhu et al., 2011), errorless motor learning has been associated with more durable skill retention (Poolton et al., 2007), and more stable movement performance, even when additional cognitive demands are introduced (El-Kishawi et al., 2021; Zareıan et al., 2015). Given these theoretical claims, researchers have increasingly examined whether the proposed benefits of errorless motor learning translate into measurable improvements in motor performance.

A few systematic reviews with varying foci have included studies of errorless motor learning. One review of motor learning paradigms in children indicated that errorless motor learning could improve movement quality and motor performance more so than error-strewn learning (van Abswoude et al., 2021). Barzyk and Gruber (2024) reviewed motor learning related to golf-specific motor skills and reported that errorless motor learning was beneficial for improving performance and accuracy among novice golfers. No systematic review has examined errorless motor learning across the lifespan thus far, despite diverse movement proficiency demands and learning abilities from childhood through older adulthood (Leversen et al., 2012). Studies in children (Capio et al., 2012; Maxwell et al., 2017), young adults (Chui et al., 2024; Fan, 2018), and older adults (Kessels and Olde Hensken, 2009; Voigt-Radloff et al., 2017) have demonstrated neurocognitive benefits from errorless motor learning, such as enhanced multitasking during skilled movements (Banihosseini et al., 2025; Poolton et al., 2005). Synthesizing these age-specific effects is crucial given developmental differences in motor and cognitive processes that influence skill acquisition and retention.

In addition to a lifespan perspective, evidence syntheses have yet to address whether errorless motor learning is a particularly suitable strategy for individuals with impairments. Such knowledge is potentially useful in neurorehabilitation, where physiotherapists adopt suitable motor learning strategies for clients with movement impairments. Kleynen et al. (2020) proposed the motor learning framework for neurorehabilitation, which identified seven strategies that include errorless motor learning. They suggested that motor learning strategies may be applied in neurorehabilitation patients while considering the elements of task organisation, feedback, and instruction. Practical application, therefore, needs to account for patient characteristics, task type, and learning stage when tailoring the intervention. Some studies have used errorless motor learning for various populations with neurological conditions and have demonstrated benefits in terms of movement outcomes (Arsham et al., 2024; Capio et al., 2013), rote learning (Kessels and Olde Hensken, 2009), and functional daily tasks (Donaghey et al., 2010; Voigt-Radloff et al., 2017). Therefore, it appears that there is potential for the use of errorless motor learning in neurorehabilitation.

To address the gaps in evidence synthesis from a lifespan perspective and rehabilitation context, the purpose of this systematic review was to synthesise the evidence on the effects of errorless motor learning on movement outcomes among children, young adults, and older adults, including those with impairments. The objectives of this study were as follows: (i) to quantify the difference in movement outcomes between errorless motor learning and control conditions; (ii) to identify age-related and impairment-related moderators; and (iii) to evaluate the methodological quality of existing evidence.

## Method

### Protocol and registration

This systematic review was developed with reference to the Preferred Reporting Items for Systematic Reviews and Meta-Analysis (PRISMA) guidelines (Page et al., 2021). The protocol was developed *a priori* and registered on the Open Science Framework (OSF) (Identifier: 10.17605/OSF.IO/SPHR6). The procedures were performed using the Covidence systematic review software.

### Search strategy

One reviewer (the first author) completed an online literature search in the following databases: MEDLINE, Embase, Cochrane Library, PubMed, CINAHL Plus, PyscINFO, Scopus, Web of Science, and PEDro. There was no restriction on the date of publication to ensure that a broader spectrum of relevant articles could be included. The citation lists of the included studies were also hand-searched to identify any relevant studies that were not previously located during the database searches. The search was performed via the following terms combined with the Boolean operators “AND” or “OR”: (errorless OR error-strewn OR error-minimising) AND (“motor learning” OR “skill acquisition”) AND performance. The first 200 results on Google Scholar and ProQuest Open Dissertation were captured to also include unpublished articles. The systematic literature search was initially conducted on October 17, 2024, and it was updated on May 1, 2025, to capture any eligible new studies published after the previous search.

### Inclusion criteria

Study selection was based on the participants, interventions, comparisons, outcomes, and study design (PICOS) framework. The framework underpinned the eligibility criteria of this systematic review and meta-analysis.

The PICOS framework was as follows:

- Participants (P): children under 18 years of age, young adults between the ages of 18 and 32, and older adults aged 60 years or above with or without disabilities. The age ranges of children and older adults were adopted from the United Nations (2002) and The UN Refugee Agency (2020), while the age definition of young adults followed the U.S. Census Bureau (2023). These three groups were deemed to reflect developmental changes across the lifespan. Middle-aged adults (aged between 32 and 59 years) were excluded from this review because relative to children, young adults, and older adults, this age group is not associated with the distinct motor and cognitive developmental changes that are central to the lifespan perspective of this review.
- Interventions (I): errorless motor learning of a discrete and/or functional task
- Comparisons (C): any motor learning of a discrete and/or functional task that allows errors to occur or accumulate
- Outcomes (O): empirical outcomes measuring process-oriented outcomes, such as movement patterns or kinematics, or product-oriented performance scores of movement stability or muscle efficiency. These outcomes measured in the acquisition or retention tests were documented. Acquisition tests assess motor performance once practice has been completed and are usually conducted immediately after the learning period. Retention tests assess motor performance after a period of having no practice. As this review aimed to explore the direct improvement and degree of motor learning, the results from transfer and secondary task tests were not included.
- Study design (S): experimental or quasi-experimental studies regardless of study setting and geographic location.

### Exclusion criteria

The following exclusion criteria were adopted: (i) non-English language studies, (ii) studies involving a serial or non-functional task, and (iii) studies in which the task did not involve a learning phase or acquisition period where participants practiced the task under designated learning environments, which could undermine the interpretation of motor learning effects.

### Selection process

The evidence was selected using a two-step screening method. Two members of the research team (first and second authors) independently screened the titles and abstracts against the eligibility criteria (level I screening). Once individual screening was completed, discrepancies were settled with a third reviewer (last author). Once the title and abstract screening was completed, full-text screening (level II screening) was independently performed by two reviewers (first and second authors). Disagreements were settled via discussion between the two researchers regarding the eligibility of the studies identified. The reference lists of the selected studies were also evaluated after level II screening, and relevant articles were identified for this review. The screening and selection processes, including reasons for exclusion, followed the PRISMA guidelines (Page et al., 2021).

### Data extraction

Once the selection of appropriate studies was completed, one researcher (first author) gathered the data using a data extraction form adapted from the Cochrane Library Data collection form (Cochrane Training, 2025), followed by secondary verification by the second reviewer (second author) to ensure accuracy and completeness. Inter-rater reliability for data extraction was assessed through independent extraction of a subset of studies, with disagreements resolved through discussion and achieving 95% agreement (calculated using percentage agreement). The information extracted from the original studies included author name(s), year of publication, study design, sample size, and participant characteristics. The types of motor tasks and details of the intervention and comparator were also extracted, as were the process-oriented and product-oriented outcomes in the form of (i) movement performance, (ii) movement accuracy, (iii) movement pattern, (iv) movement stability, (v) muscle efficiency, and (vi) kinematics.

For this review, we operationally defined movement performance as success rate or task completion – i.e., aggregated from binary or categorical outcomes indicating whether an attempt met predefined criteria (e.g., ball caught vs. not caught; movement completed within time limit). Movement accuracy was operationally defined as precision or error magnitude – i.e., a continuous measure quantifying deviation from an ideal standard (e.g., absolute error in cm from target; angular deviation in degrees from ideal trajectory). Examples of performance metrics include percentage of successful basketball free throws or proportion of correctly sequenced dance steps; examples of accuracy metrics include absolute error when reaching toward a target or temporal error during a timed tapping task. Movement pattern refers to the motion sequence and coordination of body segments when performing a task (e.g., movement pattern), whereas movement stability refers to the ability of an individual to maintain control and coordination of a motor task (e.g., jerkiness during movement). Muscle efficiency refers to the ability of muscles to produce force (e.g., muscle activation level, electromyographic activity), whereas kinematics refers to the geometric aspects of motion (e.g., position, displacement, velocity, acceleration, trajectory).

### Risk of bias and certainty assessments

Risk of bias was assessed using the risk of bias in non-randomised studies of interventions, version 2 (ROBINS-I V2) and the revised Cochrane risk of bias for randomised trials (RoB2) (Sterne et al., 2019a; Sterne et al., 2019b) tools, depending on the study design of the article. ROBINS-I V2 evaluates the risk of bias of non-randomised studies of interventions across seven bias domains. Each included study is rated as having a low, moderate, serious, or critical risk of bias on the basis of its overall rating in each bias domain. Similarly, randomised studies were assessed using RoB2 and were rated as having a low, somewhat, or serious risk of bias based on assessments in five key bias domains (Sterne et al., 2019b). The results were reviewed by the research team and finalised upon agreement. No specific level of overall bias was used as an exclusion criterion in this review. To detect the presence of publication bias in the included studies, a funnel plot was generated to examine the closeness of the estimated effect size against the true effect size (Sterne et al., 2011).

To ascertain the certainty of the evidence, the Grading of Recommendations Assessment, Development, and Evaluation (GRADE) framework (Schünemann et al., 2013) was employed as recommended by the Cochrane Collaboration. The framework encompassed risk of bias, inconsistency, indirectness, imprecision, and publication bias. One researcher (first author) undertook the ratings of evidence, whereas other members of the research team reviewed the ratings and finalised the certainty of evidence of each outcome. The certainty of evidence was rated as high, moderate, low, or very low.

### Data synthesis and analysis

A standardised data extraction form was utilised to guide the summarisation, collation, and analysis of the data for this review. Details of the study designs and demographic information of the participants, together with the movement outcomes, were analysed and discussed after the data extraction process. The PRISMA 2020 checklist (Page et al., 2021) was referenced to advise on the reporting format and items for this review.

Meta-analysis was performed using Jeffreys’s Amazing Statistics Program (JASP) version 0.19.3. Standardized mean differences were calculated using Hedges’ g, which applies a bias correction factor (J) to Cohen’s d to account for small sample sizes, computed as: g = d × [1–3/(4N − 9)], where N is the total sample size across groups. Variance for each effect size was calculated using the formula: Var(g) = (N − 2)/(N − 4) × [1/n₁ + 1/n₂ + g^2^/2(N − 2)], based on the effect sizes and sample characteristics reported by the included studies. Heterogeneity across studies was assessed using the Q-statistic and *I*^2^ index. A two-stage analytical approach was employed: first, fixed-effects models were fitted to examine summary effects under the assumption of homogeneity; subsequently, random-effects models (using restricted maximum likelihood estimation) were applied to account for between-study heterogeneity. For movement performance outcomes, a random-effects model was adopted due to significant heterogeneity detected (*I*^2^ > 50%, *p* < 0.05), reflecting meaningful variability in study designs, populations, and intervention protocols (Borenstein et al., 2021). Sensitivity analyses comparing fixed-effects and random-effects estimates were conducted to assess the robustness of findings across modelling assumptions (Cooper et al., 2019).

To prevent double-counting and violation of independence assumptions, data were restructured to ensure each study only contributed only one effect size per outcome domain to each meta-analysis. When studies reported multiple movement performance outcomes within the same domain, Hedges’ g was calculated for each outcome and combined into a single composite effect size and variance using methods outlined by Borenstein et al. (2021). For studies with multiple intervention arms sharing a common control group, the control group sample size was proportionally divided across comparisons to ensure each effect size was based on unique participant subsets. This approach guaranteed each participant was represented once only per meta-analysis and eliminate the need for additional multilevel modelling or robust variance estimation. When it was not feasible to pool the results statistically, narrative reporting was used to present the findings.

## Results

### Selection of evidence

**Figure 1:**
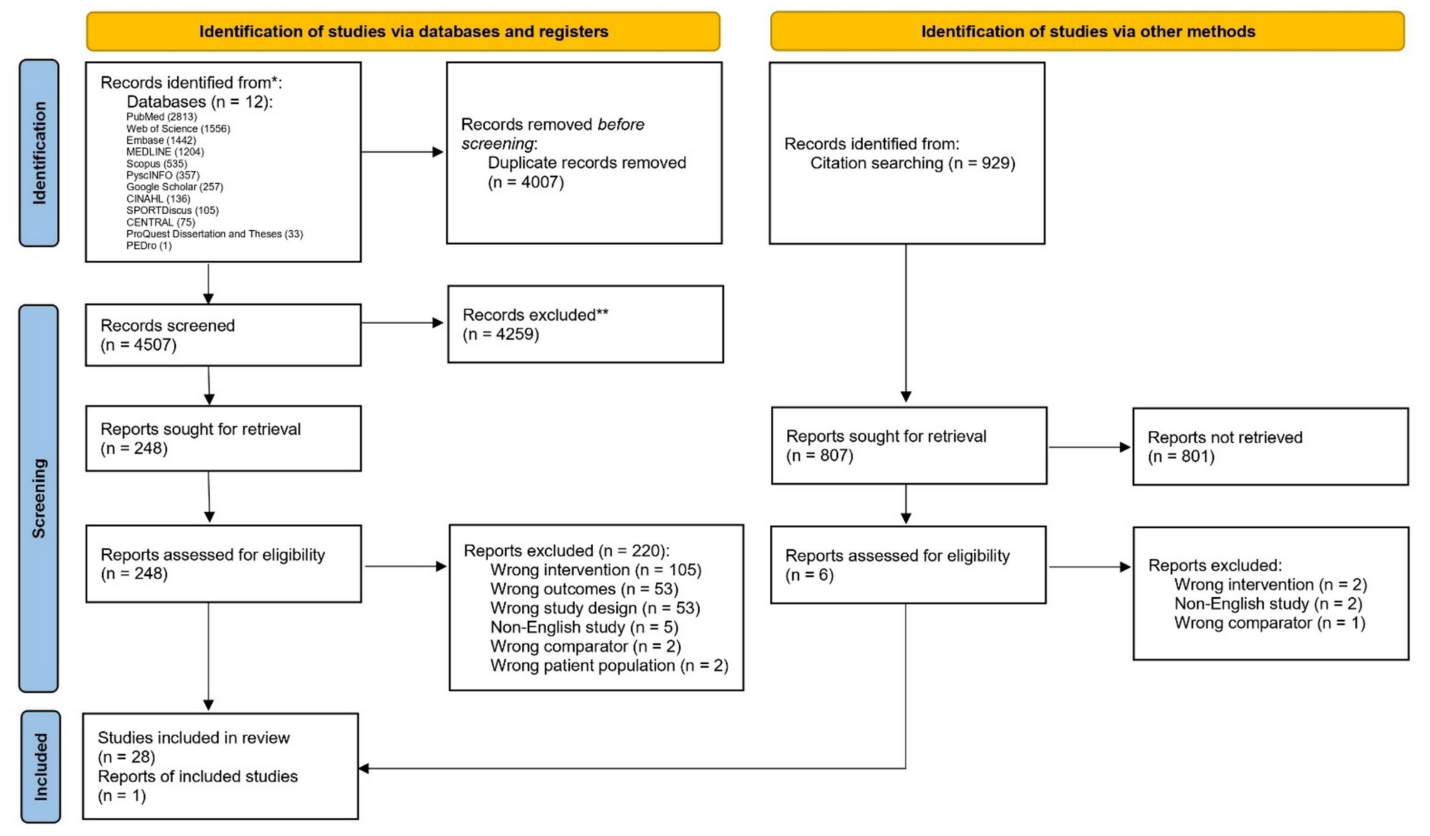
Selection of evidence based on the PRISMA flowchart (Page et al., 2021).

The PRISMA flowchart (Figure 1) illustrates the identification and screening of sources of evidence. In the identification phase, a total of 8,514 articles were identified from the search strategy, and 4,507 articles were screened after deduplication via Covidence. We further screened 248 full-text documents and ultimately included 29 articles in this review.

### Characteristics of the included studies

Settings and main findings are tabulated in Table 1. The detailed characteristics of the studies included in this review are summarised in Supplementary Table S1. Maxwell et al. (2001) and Sanli and Lee (2014) each had two eligible experiments within the same article; thus, they are labelled and discussed separately in this review (31 experiments in total).

**Table 1:** Settings and main findings of errorless motor learning.

Author (year)	Study design (sample size)	Age group	Impairment status	Task	Main findings
Abdoli et al. (2012)	Randomised trial (*n* = 30)	Young adults (22 ± 2.00 years)	None	Ball throwing	Errorless produced higher throwing precision than errorful/control: η^2^ = 0.500, *p* = 0.001 (movement accuracy)
Afrouzeh et al. (2017)	Randomised trial (*n* = 40)	Children (9.93 ± 0.55 years)	None	Basketball free throw	Errorless achieved higher free-throw scores than explicit: *t* = 17.03, *p* < 0.001 (movement performance)
Arsham et al. (2024)	Randomised trial (*n* = 20)	Children (10.15 ± 1.4 years)	Autism spectrum disorder	Golf putting	Errorless showed lower distance-from-target errors than explicit: ηp^2^ = 0.24, *p* < 0.05 (movement accuracy)
Azmı et al. (2020)	Randomised trial (*n* = 30)	Young adults (21.1 ± 1.08 years)	None	Dart throwing	Errorless yielded superior retention/transfer accuracy than errorful: d = 0.42, *p* = 0.002 (movement accuracy)
Banihosseini et al. (2025)	Randomised trial (*n* = 32)	Adults (29 ± 5.63 years)	None	Tennis ball throwing	Errorless had lower errors at retention/transfer than explicit: η^2^ = 0.30, p = 0.001 (movement accuracy)
Capio et al. (2012)	Quasi-experimental (*n* = 216)	Children (9.16 ± 0.96 years)	None	Overhead beanbag throwing	Error-reduced improved process-oriented throwing more than error-strewn: η^2^ = 0.04–0.05, p < 0.05 (movement accuracy)
Capio et al. (2013)	Quasi-experimental (*n* = 39)	Children (7.40 ± 2.10 years)	Intellectual disability	Overhead throwing	Error-reduced showed better movement form than error-strewn: η^2^ = 0.21, p < 0.05 (movement form)
Capio et al. (2017)	Quasi-experimental (*n* = 144)	Children (TD: 9.10 ± 1.10; ID: 7.20 ± 2.10 years)	Mixed (TD/ID)	Overhead throwing	Error-reduced reduced absolute error more than error-strewn: η^2^ = 0.10, p < 0.05 (movement accuracy)
Chauvel et al. (2012)	Quasi-experiment (*n* = 96)	Young (23.50 ± 3.30)/Older adults (65.00 ± 3.70 years)	None	Golf putting	Errorless increased success rates more than errorful in young adults: p < 0.05 (success rate accuracy)
Donaghey et al. (2010)	RCT (*n* = 30)	Adults (amputees)	Transtibial amputation	Prosthetic fitting	Errorless produced more correct steps than control: d = 1.25, p < 0.05 (functional steps)
Fan and Wong (2021)	Randomised trial (*n* = 36)	Older adults (71.06 ± 5.29)	None	Reaching	Errorless showed no difference in movement performance (ηp^2^ = 0.020, *p* = 0.715), lower accuracy trend (ηp^2^ = 0.158, *p* = 0.059), and better stability trend (ηp^2^ = 0.130, *p* = 0.10) vs. errorful/control.
Fan and Wong (2024)	Randomised trial (*n* = 39)	Young adults (27.03 ± 2.64)	None	Y balance lower limb reaching	Errorless showed superior movement performance (ηp^2^ = 0.184, *p* = 0.036) and stability (ηp^2^ = 0.365, p < 0.001) vs. errorful/control.
Homayounnia Firouzjah et al. (2025)	Quasi-experiment (*n* = 48)	Children (8.33 ± 0.40)	Austim spectrum disorder	Slingerball throwing	Analogy (4.0 ± 1.2) and errorless (6.1 ± 1.7) produced lower movement accuracy errors than errorful and explicit.
Khodayari et al. (2024)	Randomised trial (*n* = 20)	Children (8.63 ± 2.43)	None	Bowling	Errorless reduced movement performance equivalent to analogy (t = 0.330, *p* = 0.746) but better movement patterns (z = −2.875, *p* = 0.004).
Kleynen et al. (2019)	RCT (*n* = 56)	Older adults (64.10 ± 12.00)	Stroke	Walking	Errorless, analogy, and action observation improved walking kinematics significantly (all *p* ≤ 0.005), with errorless superior for step width.
Lam et al. (2010)	Randomised trial (*n* = 36)	Young adults (21.49 ± 2.03)	None	Golf putting	Errorless achieved movement performance equivalent to errorful (η^2^ = 0.05, *p* = 0.20) with potentially better movement stability.
Lin et al. (2023)	Randomised trial (*n* = 28)	Young adults (24.60 ± 1.60)	None	Visuo-motor (bi-rhythmic task)	Errorless improved task error accuracy over control (0.910 ± 0.265 vs. 0.690 ± 0.176), but showed reduced spectral peak accuracy (0.837 ± 0.241 vs. 1.041 ± 0.215).
Marchal–Crespo et al. (2014)	Within-group, crossover design (*n* = 22)	Young adults (23.00 ± 2.00)	None	Locomotor	All conditions (errorless, no guidance, error-amplification, heptic guidance) showed similar movement accuracy (0.024–0.04); error-amplification had highest muscle efficiency (8.75%).
Maxwell et al. (2001) _*experiment 1*	Randomised trial (*n* = 29)	Young adults (20.90 ± 2.40)	None	Golf putting	Errorless outperformed errorful and control in movement performance (t = 2.721, *p* = 0.01).
Maxwell et al. (2001) _*experiment 2*	Randomised trial (*n* = 55)	Young adults (21.00 ± 2.80)	None	Golf putting	Errorless outperformed errorful in movement performance (40 vs. 25) but showed better movement accuracy in experimental conditions (18 vs. 90).
Maxwell et al. (2017)	Randomised trial (*n* = 45)	Children (9.80 ± 0.59)	None	Golf putting	Errorless produced consistent movement performance across ability levels (high: 50 ± 5, low: 49 ± 5), while errorful varied widely (high: 58 ± 4, low: 43 ± 5).
Mount et al. (2007)	Randomised controlled trial (crossover) (*n* = 33)	Adults (63.00 ± 12.00 years)	Stroke	Wheelchair preparation and sock-donning	Errorless showed higher retention rates in impaired memory groups than trial-and-error (movement performance)
Orrell et al. (2006a) – *healthy subjects*	Quasi-experiment (*n* = 36) years	Young adults (20.29 ± 1.17)	None	Balancing	No significant group effect on RMSE: Wilk’s Λ = 0.968, *p* = 0.58 (movement accuracy)
Orrell et al. (2006b) – *stroke patients*	Quasi-experiment (*n* = 22)	Adults (52.17–65.25 years)	Stroke	Dynamic balancing	No significant group effect on RMSE: *F* = 2.39, p = 0.10 (movement accuracy)
Poolton et al. (2005)	Randomised trial (*n* = 35)	Young adults (21.10 ± 1.48 years)	None	Golf putting	No significant group effect on successful putts: η^2^ = 0.002, *p* = 0.79 (movement performance)
Poolton et al. (2007)	Randomised trial (*n* = 55)	Young adults (23.00 ± 4.95 years)	None	Rugby passing	No significant group effect on accuracy: η^2^ = 0.002, *p* = 0.76 (movement accuracy)
Ramezanzade et al. (2022)	Randomised trial (*n* = 120)	Young adults (21.19 ± 1.40 years)	None	Dart throwing	Random-errorless produced lower performance error than others: ηp^2^ = 0.662, p = 0.001 (movement accuracy)
Sanli and Lee (2014) -*experiment 1*	Randomised trial (*n* = 19)	Adults (25.60 ± 3.20 years)	None	Aiming	No significant difference in proportion of errors: p > 0.05 (movement accuracy)
Sanli and Lee (2014) - *experiment 2*	Randomised trial (*n* = 20)	Young adults (21.20 ± 2.90 years)	None	Aiming	No significant difference in proportion of errors: *p* > 0.05 (movement accuracy)
Savelsbergh et al. (2012)	Quasi-experiment (*n* = 40)	Young adults (20.50 ± 0.70 years)	None	Soccer free-kicking	Errorless produced higher free-kick points than others: η^2^ = 0.12, p < 0.05 (movement performance)
van Abswoude et al. (2015)	Quasi-experiment (*n* = 38)	Children (9.50 ± 4.00 years)	Cerebral palsy	Aiming	Error-minimizing produced higher errors during practice than error-strewn (movement accuracy)

The included studies recruited 1,509 participants, with the reported mean age of participants ranging from 7.22 to 71.06 years across the studies. Publications were released between 2001 and 2025. Seven experiments were conducted between 2001 and 2009, 15 between 2010 and 2019, and nine between 2020 and 2025. Most experiments were published in Hong Kong (9/31, 29.03%), followed by Iran (7/31, 22.58%), the United Kingdom (5/31, 16.13%), and the Netherlands (3/31, 9.68%). Eighteen experiments investigated the effects of errorless motor learning on movement performance, 17 on movement accuracy, five on movement stability, two on kinematics, two on movement patterns, and one on muscle efficiency. The tasks ranged from discrete tasks such as robotic golf putting and dart throwing to functional tasks such as walking and prosthetic limb fitting.

The demographics of the participants also varied: 10 experiments recruited children, 17 experiments recruited young adults, and five experiments recruited older adults. Chauvel et al. (2012) recruited both young and older adults in the same experiment, which accounted for the duplication. Among these participants, 10 experiments included those with impairments, including stroke (*n* = 3), autism spectrum disorder (ASD) (*n* = 3), intellectual disability (*n* = 2), unilateral transtibial amputation (*n* = 1), and cerebral palsy (*n* = 1).

### Synthesis of results

#### Movement performance

Fourteen experiments (Abdoli et al., 2012; Afrouzeh et al., 2017; Banihosseini et al., 2025; Capio et al., 2012; Capio et al., 2013; Capio et al., 2017; Donaghey et al., 2010; Fan and Wong, 2024; Khodayari et al., 2024; Lam et al., 2010; Maxwell et al., 2001; Poolton et al., 2005, 2007) assessed movement performance, where Capio et al. (2017) examined typically developed children and children with intellectual disability separately, involving 14 experimental groups and 14 comparison groups (pooled *n* = 737). Three studies – Abdoli et al. (2012) and Banihosseini et al. (2025) on ball throwing, and Capio et al. (2012, 2013, 2017) on overhead throwing – utilised various throwing tasks. Others focused on golf putting (Lam et al., 2010; Maxwell et al., 2001; Poolton et al., 2005), basketball free throws (Afrouzeh et al., 2017), prosthetic limb fitting (Donaghey et al., 2010), balance and lower limb reaching (Fan and Wong, 2024), bowling (Khodayari et al., 2024), and rugby passing (Poolton et al., 2007). The meta-analysis, as illustrated in Supplementary Figure S1, revealed that the effect of errorless compared to errorful motor learning on movement performance was not significant. The full-sample random-effects model yielded *g* = 0.506 (95% CI = −0.308 to 1.319; *z* = 1.18; *p* = 0.223). High heterogeneity was observed [Q(13) = 115.50; *p* < 0.001; *I*^2^ = 96.062%; τ^2^ = 2.265] with a wide prediction interval (−2.54 to 3.57). Under a fixed-effects model, g = 0.487 (95% CI: 0.303 to 0.672). Sensitivity analysis excluding one high risk of bias study identified via ROBINS-I (*k* = 12) yielded a random-effects pooled *g* = 0.491 (95% CI: −0.474 to 1.460; z = 0.994, *p* = 0.320; Q = 113.80, df = 11, *p* < 0.001). Heterogeneity remained high after excluding this study, indicating that its removal neither resolved between-study variability nor materially reduced the pooled random-effects estimate. The direction of the effect remained positive and broadly consistent across fixed-effect and random-effects models, but the wide confidence and prediction intervals and substantial heterogeneity indicate considerable uncertainty, warranting further exploration of potential sources of bias and heterogeneity.

Among the 14 experiments included, six were conducted on children (Afrouzeh et al., 2017; Capio et al., 2012; Capio et al., 2013; Capio et al., 2017; Khodayari et al., 2024), seven were conducted on young adults (Abdoli et al., 2012; Banihosseini et al., 2025; Fan and Wong, 2024; Lam et al., 2010; Maxwell et al., 2001; Poolton et al., 2005; Poolton et al., 2007), and one focused on older adults (Donaghey et al., 2010). As shown in Supplementary Figure S2, the effect of errorless motor learning on movement performance of children was not significant (*g* = 1.013; 95% CI = −0.074 to 2.100; *z* = 1.83; *p* = 0.068) when compared to errorful learning condition, and high heterogeneity was observed across the studies [Q(5) = 47.59; *p* < 0.001; *I*^2^ = 95.996%; τ^2^ = 1.719] with a broad prediction interval (−1.78 to 3.80). In young adults (Supplementary Figure S3), the effect of errorless compared to errorful motor learning on movement performance was not significant (*g* = −0.063; 95% CI = −1.375 to 1.249; *z* = −0.09; *p* = 0.925) and heterogeneity was significant [Q(6) = 61.11; *p* < 0.001; *I*^2^ = 95.711%; τ^2^ = 3.890] with a wide prediction interval (−3.69 to 3.56). No analysis was performed on older adults, as there was only one study (Donaghey et al., 2010), which reported that the errorless group performed better than the control group after the intervention.

**Figure 2:**
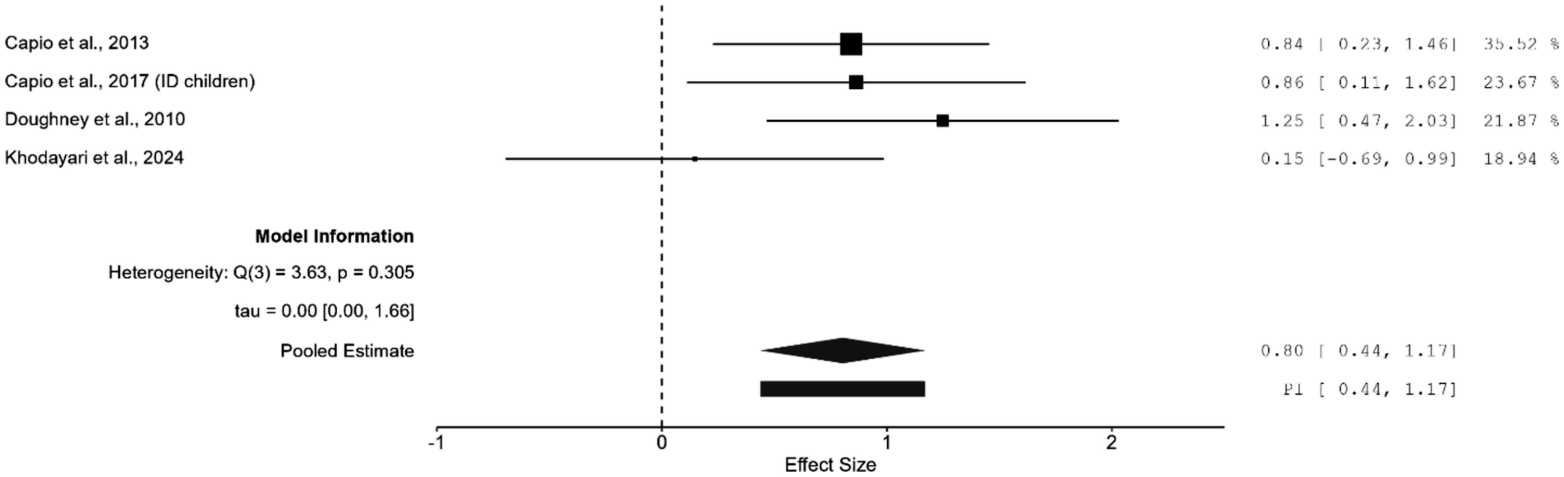
Subgroup analysis of the effect of errorless motor learning on movement performance among learners with impairment.

Subgroup analysis on the effect of errorless compared to errorful motor learning on people with or without impairment was also carried out. Among the four studies that included people with impairments (Capio et al., 2013; Capio et al., 2017; Donaghey et al., 2010; Khodayari et al., 2024), a significant large effect was observed (*g* = 0.804; 95% CI = 0.438 to1.170; *z* = 4.31; *p* < 0.001) as shown in Figure 2. Variability was not significant [Q(3) = 3.63; *p* = 0.305; *I*^2^ = 1.768 × 10^−4^%; τ^2^ = 2.67 × 10^−7^] with a narrow prediction interval (0.44 to 1.17). Ten studies investigated the effect of errorless compared to errorful motor learning on people without impairment (Abdoli et al., 2012; Afrouzeh et al., 2017; Banihosseini et al., 2025; Capio et al., 2012; Capio et al., 2017; Fan and Wong, 2024; Lam et al., 2010; Maxwell et al., 2001; Poolton et al., 2005, 2007), and the results revealed no significant effect (*g* = 0.392; 95% CI = −0.775 to 1.560; *z* = 0.66; *p* = 0.510) (Supplementary Figure S4); significant heterogeneity was observed [Q(9) = 106.96; *p* < 0.001; *I*^2^ = 97.595%; τ^2^ = 3.396] with a wide prediction interval (−3.41 to 4.19).

#### Movement accuracy

**Figure 3:**
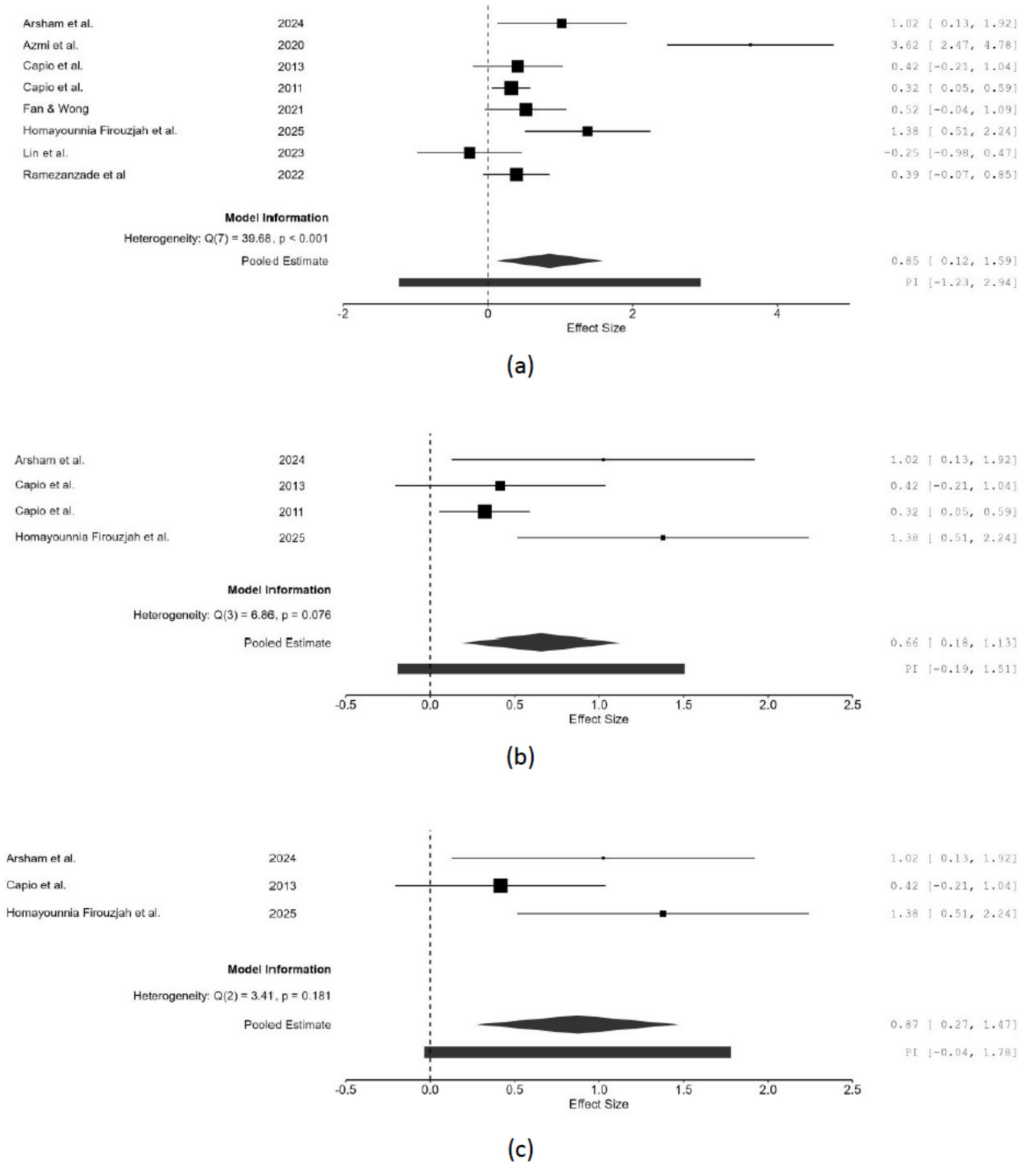
Effects of errorless motor learning on movement accuracy overall **(a)**, among children **(b)**, and among learners with impairment **(c)**.

Eight studies, consisting of eight experimental groups and eight comparators with a pooled *n* = 513 (Arsham et al., 2024; Azmı et al., 2020; Capio et al., 2013; Capio et al., 2012; Fan and Wong, 2021; Homayounnia Firouzjah et al., 2025; Lin et al., 2023; Ramezanzade et al., 2022), examined movement accuracy. Two studies each examined dart throwing (Azmı et al., 2020; Ramezanzade et al., 2022), and overhead throwing (Capio et al., 2013; Capio et al., 2012). One study each investigated reaching (Fan and Wong, 2021), slingerball throwing (Homayounnia Firouzjah et al., 2025), and visuo-motor bi-rhythmic task (Lin et al., 2023). The direction of effect size in four studies was adjusted to ensure consistency in reporting the results. As illustrated in Figure 3, the full-sample random-effects model revealed a significant moderate effect of errorless relative to errorful motor learning on movement accuracy (*g* = 0.854; 95% CI = 0.119 to1.589; *z* = 2.28; *p* = 0.023). Significant heterogeneity was observed [Q(7) = 39.68; *p* < 0.001; *I*^2^ = 92.902%; τ^2^ = 0.990] with a broad prediction interval (−1.23 to 2.94). Under a fixed effects model, *g* = 0.487, (95% CI: 0.303 to 0.672, *p* < 0.001). Sensitivity analysis excluding two high/serious risk of bias studies identified via RoB2 and ROBINS-I (*k* = 6) yielded a random-effects g = 0.358 (95% CI: 0.167 to 0.549, *z* = 3.672, *p* < 0.001; *Q* = 5.326, *p* = 0.377; *I*^2^ = 7.983 × 10^−4^%; τ^2^ = 5.525 × 10^−7^). The effect size attenuated substantially (Δg = −0.496, 58% reduction), with heterogeneity fully resolved (non-significant *Q*-test, *I*^2^ ≈ 0%), suggesting studies with high/serious risk of bias inflated the effect size. There remained a small but significant effect which appears consistent across the better-quality studies.

Subgroup analyses were performed on different age groups to investigate the effects of errorless motor learning in each age group. Among the eight studies included, four were conducted on children (Arsham et al., 2024; Capio et al., 2012; Capio et al., 2013; Homayounnia Firouzjah et al., 2025), three were on young adults (Azmı et al., 2020; Lin et al., 2023; Ramezanzade et al., 2022), and one was on older adults (Fan and Wong, 2021). As illustrated in Figure 3, errorless motor learning had a significant moderate effect on movement accuracy among children (*g* = 0.657; 95% CI = 0.1183 to 1.131; *z* = 2.72; *p* = 0.007) when contrasted with errorful learning condition, and heterogeneity was not significant [Q(3) = 6.86; *p* = 0.076; *I*^2^ = 58.187%; τ^2^ = 0.130] with a narrow prediction interval (−0.19 to 1.51). The effect of errorless motor learning on young adults was not significant (*g* = 1.210; 95% CI = −1.092 to 3.512; *z* = 1.03; *p* = 0.303) as opposed with errorful learning condition as shown in Supplementary Figure S5, and significant heterogeneity was found [Q(2) = 32.58; *p* < 0.001; *I*^2^ = 96.655%; τ^2^ = 3.962] with a wide prediction interval (−3.20 to 5.62). There was only one study that recruited older adults; thus, no analysis was performed for this age group.

In the three studies investigating individuals with impairments (Arsham et al., 2024; Capio et al., 2013; Homayounnia Firouzjah et al., 2025), subgroup analysis revealed a significant large effect of errorless compared to errorful motor learning (*g* = 0.873; 95% CI = 0.273 to1.472; *z* = 2.85; *p* = 0.004) (Figure 3), and heterogeneity was not significant [Q(2) = 3.42; *p* = 0.181; *I*^2^ = 42.834%; τ^2^ = 0.121] with a narrow prediction interval (−0.04 to 1.78). Among the five studies that included people without impairments (Azmı et al., 2020; Capio et al., 2012; Fan and Wong, 2021; Lin et al., 2023; Ramezanzade et al., 2022), the subgroup analysis (Supplementary Figure S6) revealed no significant effect of errorless compared to errorful motor learning (*g* = 0.859; 95% CI = −0.388 to2.106; *z* = 1.35; *p* = 0.117), and significant heterogeneity was observed [Q(4) = 33.75; *p* < 0.001; *I*^2^ = 96.475%; τ^2^ = 1.903] with a wide prediction interval (−2.12 to 3.89).

#### Movement stability

Four studies reported the effects of errorless motor learning on movement stability variables (Fan and Wong, 2021; Fan and Wong, 2024; Lam et al., 2010; Poolton et al., 2005). These four studies reported various aspects of movement stability outcomes, including jerkiness of acceleration (Fan and Wong, 2021; Fan and Wong, 2024), number of visual adjustments (Lam et al., 2010), and number of technique changes (Poolton et al., 2005), which precludes meaningful combination of the results in a meta-analysis, as they represented various aspects of movement stability. Two studies (Fan and Wong, 2021; Fan and Wong, 2024) reported the jerkiness of acceleration in reaching motor tasks and dynamic balance tasks. A significant effect (*p* < 0.001) of errorless compared to errorful motor learning on the jerkiness of acceleration was found for the dynamic balance task (Fan and Wong, 2024), whereas there was no significant effect (*p* = 0.10) on the reaching task outcome (Fan and Wong, 2021). The remaining two studies (Poolton et al., 2005; Lam et al., 2010) reported the number of technique/visual changes in a golf-putting task. Poolton et al. (2005) reported a low level of technique changes in the errorless group, whereas Lam et al. (2010) reported few visual changes in the errorless group in the learning phase. However, no statistical comparisons of the number of technique changes between errorless and errorful learning groups were conducted.

#### Movement pattern

Two studies assessed the effects of errorless motor learning on movement patterns in children with and without intellectual disability (Capio et al., 2017) and in children with ASD (Khodayari et al., 2024). Capio et al. (2017) reported improvements in movement patterns for both errorless and errorful learning groups, and across typically developing children (*n* = 108) and children with intellectual disability (*n* = 36). The benefit of errorless motor learning, however, was only significant for children with intellectual disability (*p* = 0.006). Khodayari et al. (2024) also reported that analogy learning with external focus was more beneficial than errorless learning with external focus for underhand throw movement patterns (*p* = 0.004, *z* = 2.88) among children with ASD (*n* = 20).

#### Muscle efficiency

One study (Marchal–Crespo et al., 2014; *n* = 22) investigated the effect of errorless motor learning on muscle efficiency in young adults when they learned a locomotor task using robotic training that enforced desired knee trajectory of participants by manipulating the knee cylinder during the experiment. The study revealed significantly greater muscle activation in the comparator group than in the errorless group in the first third of the retention test (*p* = 0.011), but the difference was not significant toward the last third of the retention test (*p* = 0.075).

#### Kinematics

Two studies (Kleynen et al., 2019; Arsham et al., 2024) assessed the effects of errorless motor learning on kinematics outcomes. Arsham et al. (2024) reported golf putting kinematics in children with ASD (*n* = 20), comparing errorless training to explicit instruction. On retention testing, no kinematic differences emerged between groups for putter face angle, backswing duration, backswing-to-impact timing, or impact-to-follow-through phase. Kleynen et al. (2019) investigated immediate spatiotemporal gait kinematics in post-stroke patients (*n* = 56) using implicit strategies including errorless learning by constraining the environment using narrow beams. Errorless learning was found beneficial for improving step width (*p* < 0.001).

### Risk of bias in individual studies and certainty of evidence

RoB2 assessments were employed in 20 randomised trials, whereas 10 non-randomised trials were evaluated via ROBINS-I assessments. Supplementary Figures S7–S9 present the results of the RoB2 and ROBINS-I assessments. Among the 31 experiments, 24 experiments had an overall moderate risk of bias or some concerns, six experiments had a serious (or high) risk of bias, and only one trial had a low risk of bias. Among the 20 randomised trials, only two reported their randomisation sequence generation method, which involved blind labelling and block randomisation by a blind assessor. The remaining randomised trials did not describe or specify their randomisation processes.

**Figure 4:**
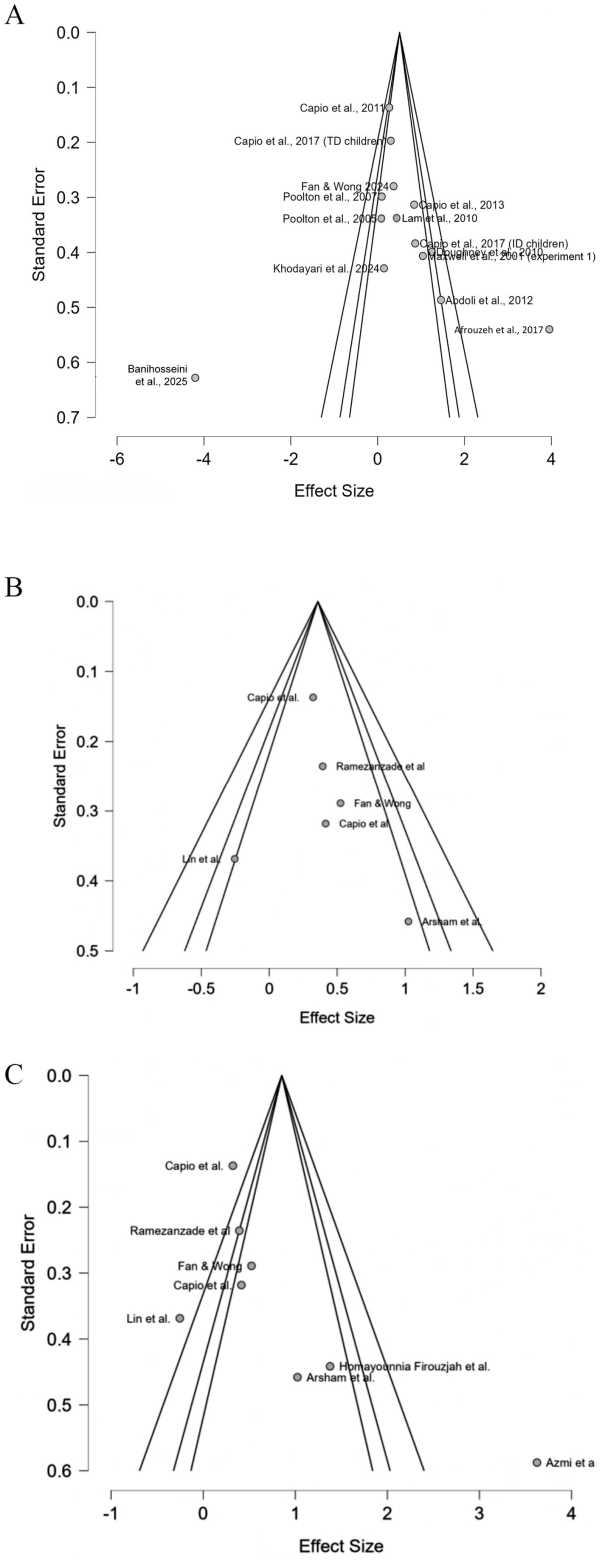
Funnel plots for publication bias assessment: **(A)** movement performance, **(B)** movement accuracy, **(C)** movement accuracy (high-risk studies removed).

As shown in Figures 4A,B, visual inspection of the funnel plots indicated possible publication bias. To further evaluate this, Egger’s regression test was performed. No statistically significant evidence of publication bias was found for movement performance (Egger’s *z* = −0.384; *p* = 0.071; weighted regression *t* = 0.530; df = 12; *p* = 0.606; Begg’s *τ* = 0.385; *p* = 0.062), while there is a statistically significant indication of publication bias for movement accuracy (Egger’s *z* = 2.696; *p* = 0.007; 95% CI = −2.536 to 0.383). The removal of two high/serious risk of bias experiments (Figure 4C) on movement accuracy yielded Egger’s z of 0.456 (*p* = 0.648; weighted regression *t* = 0.403; df = 4; *p* = 0.707), supporting funnel symmetry around the homogeneous pooled *g* = 0.358. With heterogeneity resolved, these results suggest minimal selective reporting risk, supporting the reliability of the conservative effect estimate.

Table 2 displays the results of the GRADE analysis. The certainty in the evidence of outcomes from the GRADE analysis was found to be very low to low. All the outcomes were found to pose a serious risk of bias; two had serious inconsistency and publication bias. Movement pattern, muscle efficiency, and kinematics each had one to two studies in their category, which affected the overall grading of the certainty of their evidence.

**Table 2:** GRADE approach: evidence profile.

Certainty assessment								
Outcome	No. of studies	Risk of bias	Inconsistency	Indirectness	Imprecision	Publication bias	Other considerations	Certainty
Movement performance	14	Serious	Not serious	Not serious	Not serious	Undetected	None	⊕ ⊕ ◯◯
Movement accuracy	8	Serious	Serious	Not serious	Not serious	Serious	None	⊕ ⊕ ◯◯
Movement stability	5	Serious	Serious	Not serious	Not serious	Serious	None	⊕ ⊕ ◯◯
Movement pattern	2	Serious	Unaccessable	Not serious	Not serious	Unaccessable	Only 2 studies available	⊕◯◯◯
Muscle efficiency	1	Serious	Unaccessable	Not serious	Not serious	Unaccessable	Only 1 study available	⊕◯◯◯
Kinematics	2	Serious	Unaccessable	Not serious	Not serious	Unaccessable	Only 2 studies available	⊕◯◯◯

## Discussion

To the best of our knowledge, this is the first systematic review and meta-analysis to examine the effects of errorless motor learning on movement outcomes with consideration for age groups and impairments. In terms of the demographics of the included studies, more than half of the participants were young adults and people without impairments, accounting for more than half of the population reviewed. Nevertheless, our sub-group analyses allowed us to delineate the evidence from a lifespan perspective and with consideration for impairments.

From the meta-analyses of the movement performance outcomes compared with control conditions, there appears to be no overall advantage afforded by errorless motor learning. With the reviewed studies reflecting high heterogeneity, there is likely substantial variability in tasks, populations and implementation of the interventions. While the calculated overall effect size indicates a medium effect, the confidence interval being relatively wide suggests significant variation across studies. The studies reported on experiments that spanned different types of motor skills and outcome measures, as well as diverse learner groups, among whom the relative benefit of minimising errors versus learning from them may differ. Moreover, practice conditions were operationalised inconsistently across studies (e.g., degree of error manipulation, progression of task difficulty), which may have weakened the contrast between conditions. Given such heterogeneity in evidence, the overall advantage of errorless motor learning on movement performance is not supported.

There appears to be an overall advantage of errorless motor learning for movement accuracy, which tended to be measured across studies more consistently. With errorless motor learning considered to fall within the implicit motor learning paradigm (Kleynen et al., 2020; Maxwell et al., 2001), facilitating accurate movement outcomes while minimising the interference of errors in the early stage of practice. Thus, it could be associated with reduced cognitive load on learners and limited competition for motor memories caused by making mistakes, enhancing skill acquisition in a more accurate manner (Poolton et al., 2007). Our meta-analysis supports these theoretical propositions for errorless motor learning given improved movement accuracy.

From a lifespan perspective, errorless motor learning appeared beneficial for movement accuracy only in children, which we suggest to be likely linked to the development of motor and cognitive systems in childhood. Increased neuroplasticity is expected in childhood (Weyandt et al., 2020). We speculate that success during practice could reinforce neural connections for accurate motor skills while limiting the formation of incorrect ones (Arsham et al., 2024; Capio et al., 2012), but which needs verification in future research. From a working memory perspective, errorless motor learning could allow children to focus their developing cognitive resources (Gathercole et al., 2004) on aiming for accurate outcomes rather than processing errors. As executive functions, working memory, and other cognitive functions develop during childhood, success during practice likely allows children to execute movements without cognitive overload (Capio et al., 2024). Frequent success through successful practice experiences also fosters confidence and engagement among child learners (Wulf and Lewthwaite, 2016). Moreover, children appear to be particularly well suited to developing automatic and precise movements without overthinking and excessive conscious control (Capio et al., 2013; Schmidt and Lee, 2011).

In contrast, errorless motor learning among young adult learners does not appear superior to control conditions in either movement performance or movement accuracy. Young adults with fully developed cognitive functions and motor control are likely to benefit less from errorless motor learning because errors provide essential feedback for them to adapt and refine their motor skills (Lee et al., 2016; Maxwell et al., 2001). Conscious awareness of errors has been suggested to be important in refining motor performance in adult learners (Seidler et al., 2013a,b; Truong et al., 2023), which allows them to compare their actual performance with their predicted performance and optimise movement control using the discrepancies (Truong et al., 2023).

For older adults, there was only one study, each for movement performance and accuracy, reflecting limited research in this particular population. While the age-related decline in cognitive functions of older adults suggest errorless motor learning to be suitable for this demographic, earlier research has suggested that older adults tend to rely more on explicit and error-based learning with seemingly reduced implicit learning capacity (Howard Jr and Howard, 1997). Moreover, motor learning studies of older adults tend to focus on functional tasks that directly translate into daily life, such as those related to balance and gait (Donaghey et al., 2010; Fan and Wong, 2021; Mount et al., 2007; Orrell et al., 2006a). Errorless motor learning may not be a straightforward approach for such functional tasks because it requires breaking down a motor task into increments of difficulty levels. Such complexity in adapting errorless paradigms for balance and gait tasks could have drawn researchers’ interest to other motor learning approaches that better align with manipulating and designing such tasks. Nevertheless, we propose that future studies could examine errorless motor learning for older adults given the likely suitability of cognitive demands with the developmental changes in late adulthood.

From an impairment perspective, the available evidence suggest that errorless motor learning is potentially beneficial for learners with impairments both in terms of movement performance and accuracy, which is not the case for learners without impairments. Learners with impairments are likely to benefit from a simplified and guided learning environment to compensate for their motor and cognitive deficits (Boyd and Winstein, 2004). Conceptually, errorless motor learning meets their needs through a clear and consistently guided learning environment that promotes accurate movement patterns, relevant to rehabilitation, as suggested by Steenbergen et al. (2010). The present findings, however, offer limited and preliminary scientific basis for the application of errorless motor learning in neurorehabilitation given the non-clinical outcomes (e.g., throwing skills of children). Future research could explore implementation science approaches, supported by the framework described by Kleynen et al. (2020), to examine the application of errorless motor learning in clinical neurorehabilitation contexts particularly among children. An implementation case study has suggested the suitability of errorless motor learning for object control skills of children with intellectual disability (Capio and Eguia, 2021).

### Limitations and strength of evidence

While this review offers evidence for the benefits of errorless motor learning in improving movement outcomes among children and those with impairments, several limitations exist. Only a small number of randomised controlled trials (RCTs) were included, which limits the strength of evidence. Additionally, intervention protocols using an errorless motor learning approach, as well as their evaluation methods, varied across studies, hindering the comparability of the results. The included experiments in this review did not adopt the contemporary methodological standards and standardized toolboxes/frameworks that set the ground for transparent and reproducible motor learning research. The omission of validated and standardised protocol such as Brain Electrophysiological recording & STimulation (BEST) toolbox (Hassan et al., 2022) likely contributed to variability in how errorless learning interventions were conducted and subsequently accounted for the substantial heterogeneity in meta-analyses and low certainty of evidence observed in the GRADE assessments. The most significant limitation is the low certainty of evidence, with all findings rated with low to very low certainty of evidence. There appears substantial uncertainty of evidence due to serious risks of bias, unexplained heterogeneity, imprecision from small sample size, and potential publication bias. Therefore, considering the limited certainty in evidence, clinical practitioners are recommended to view errorless motor learning as a potentially beneficial approach at this stage. Errorless motor learning could be implemented judiciously in training programs with careful monitoring until stronger and higher quality evidence accumulates. Nevertheless, we note that the majority of the studies were published more than a decade ago, during which publications were less obliged to meet standard reporting guidelines, limiting consistency in transparent reporting (Plint et al., 2006; Li et al., 2024). It is possible that the assessed strength of evidence could be undermined by this lack of detailed reporting in earlier publications.

Given the potential for application in neurorehabilitation, the current limitations underscore the need for more rigorous experiments on the topic to generate confidence in applying this paradigm in clinical contexts. The lack of studies conducted in older adults also limits the basis for conclusions related to older adults, whose cognitive decline requires learning strategies that are less demanding of their cognitive resources. Moreover, the absence of long-term follow-up data in most of the studies leaves the matter of long-term retention of motor skills largely uncertain. More high-quality studies, especially RCTs, are needed to corroborate these findings in the future.

We also acknowledge limitations of this review. As only English articles were included, relevant studies published in other languages were potentially missed from this review. The included experiments also varied in their protocols and reported outcomes, making it difficult to synthesise the evidence of some outcomes quantitatively, which may affect the precision in the estimates of effectiveness of errorless motor learning in these outcomes. Furthermore, the search strategy of this current review did not include trial registry searches, and the search terms were errorless-focused, hence it is recommended that future reviews incorporate supplementary searches using relevant concepts such as guided learning and error-minimisation to capture more pertinent studies.

This review synthesised evidence across a wide spectrum of motor tasks, including discrete tasks (e.g., dart throwing and golf putting) and functional tasks (e.g., walking and prosthetic limb fitting), Discrete tasks have clear beginnings and ends with close-loop feedback while functional tasks engage learners with feed-forward adaptability. The absence of subgroup analyses due to sparse data per category, makes it difficult to confidently generalise the effects of errorless motor learning clinically relevant daily activities. Future studies are recommended to stratify task types to differentiate outcomes of discrete versus functional tasks. Finally, because we focused on the direct improvement of motor outcomes, data from transfer and secondary task tests were not included in the meta-analysis. This may narrow the scope of results to the more immediate and task-specific effects of errorless motor learning in contrast to errorful learning condition and underestimate its potential benefits on skill adaptability especially in novel and cognitively challenging conditions. We also cannot rule out that an evidence synthesis focused on transfer tests may reveal a different pattern in relation to age and impairment.

## Conclusion

The findings from this systematic review and meta-analysis suggest that errorless motor learning could potentially enhance movement performance and accuracy in children and among those with impairments. While the majority of the reviewed studies demonstrated that errorless motor learning is beneficial to learners, the strength of the available evidence is insufficient due to weaknesses in methodological quality of the reviewed studies. While there is an evident potential value to adapting errorless motor learning for children and clinical applications, there needs to be cautious interpretation of evidence and future studies with robust designs are recommended.

## Data Availability

The original contributions presented in the study are included in the article/Supplementary material, further inquiries can be directed to the corresponding author.
