# Supplementary material for: A systematic review and meta-analysis of the effects of errorless motor learning on movement outcomes: a lifespan and impairment perspective
Source: Front Psychol. 2026 Mar 24;17:1722743. doi: 10.3389/fpsyg.2026.1722743 (PMC13053278; doi:10.3389/fpsyg.2026.1722743)
Supplement: Supplementary file 1 [file Table_1.DOCX]

Supplementary Figures


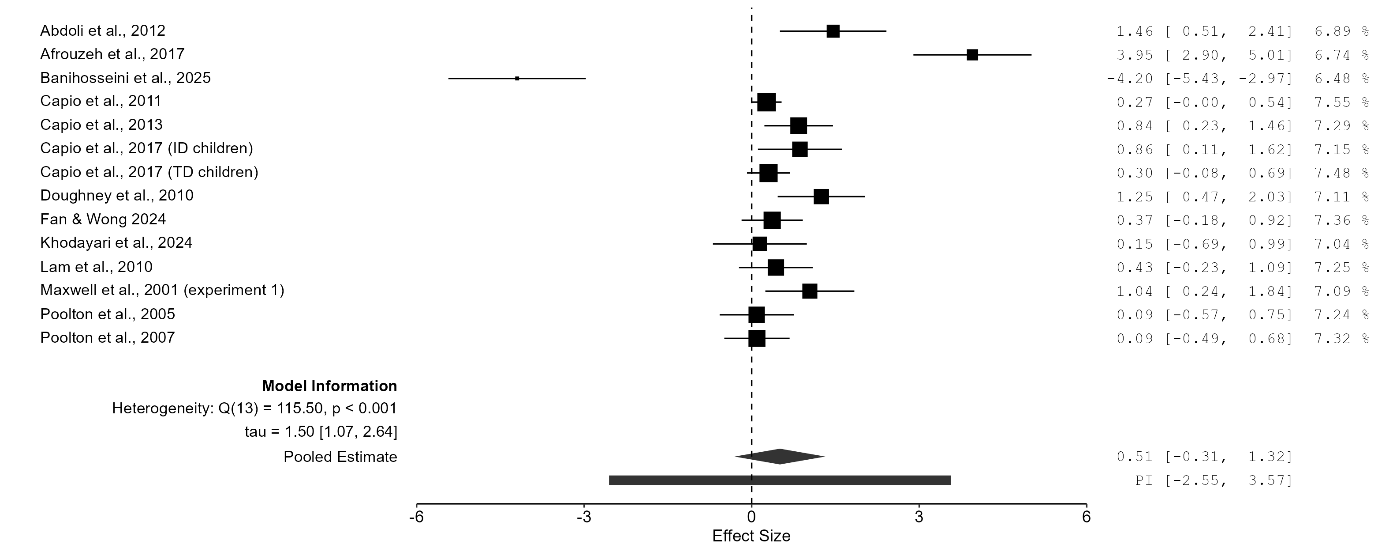


Figure S1. Overall effect of errorless motor learning on movement performance.


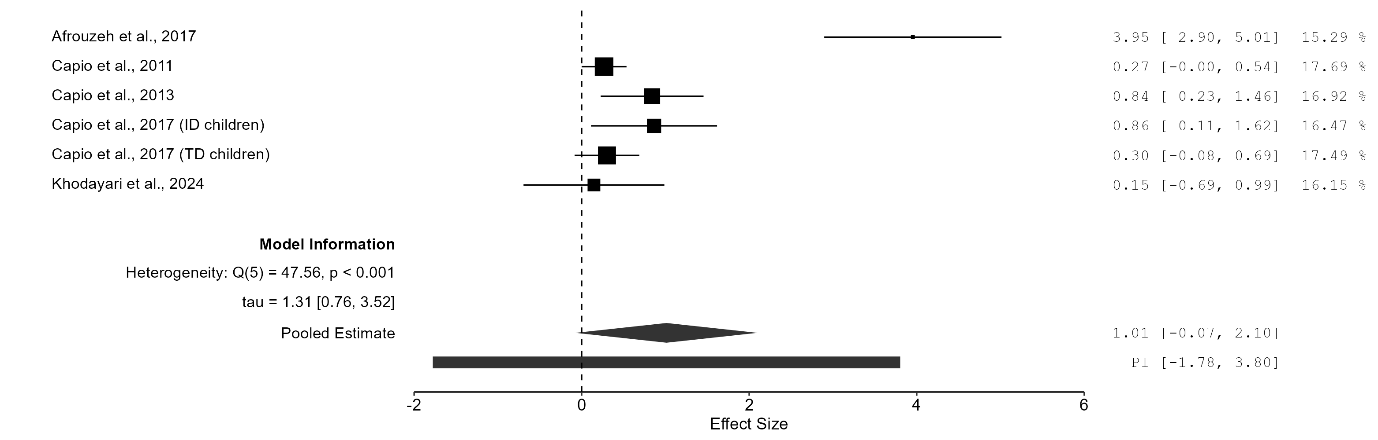


Figure S2. Effect of errorless motor learning on movement performance among children.


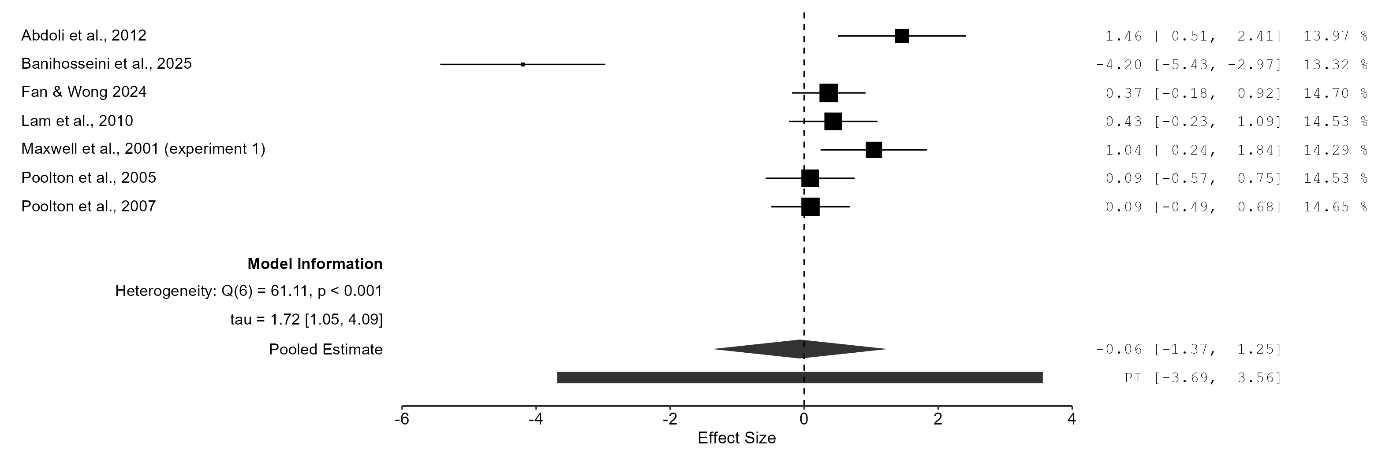


Figure S3. Effect of errorless motor learning on movement performance among young adults.


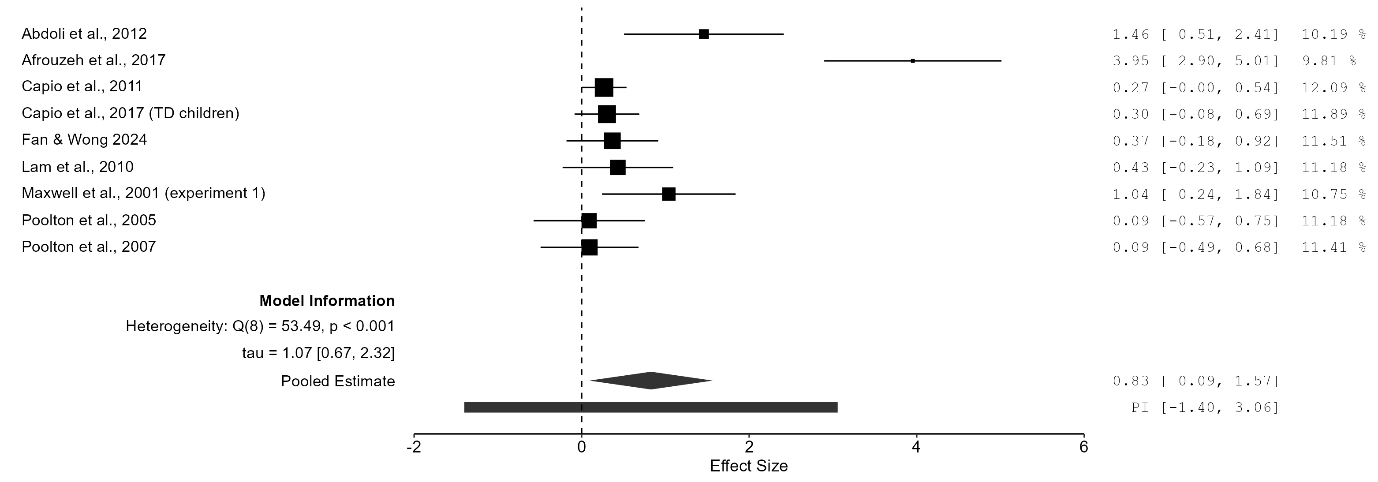


Figure S4. Effect of errorless motor learning on movement performance among learners without impairments.


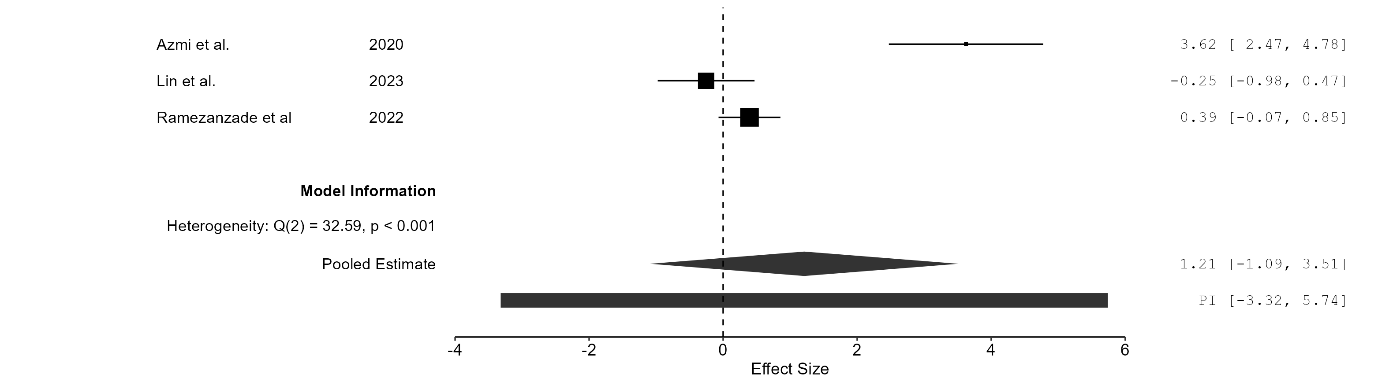


Figure S5. Effect of errorless motor learning on movement accuracy among young adults.


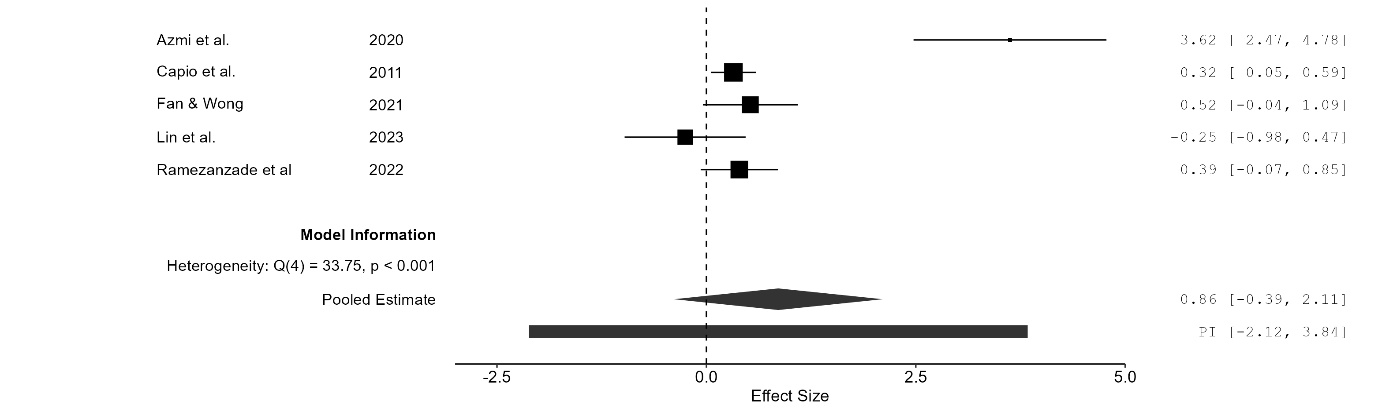


Figure S6. Effect of errorless motor learning on movement accuracy among learners without impairments.


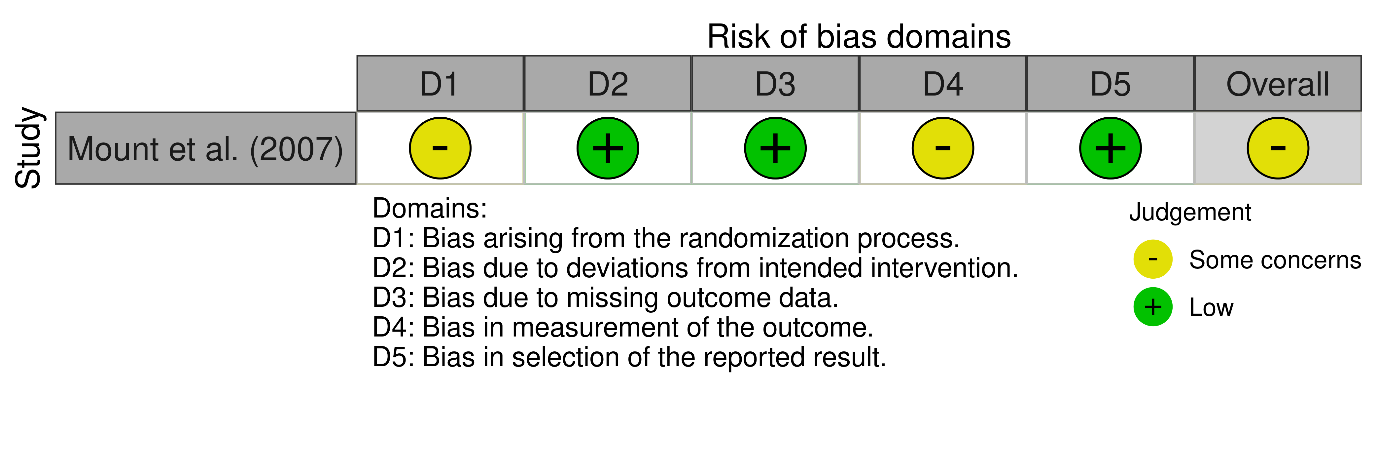


Figure S7. Risk of bias assessment of a randomised crossover study (RoB-2).


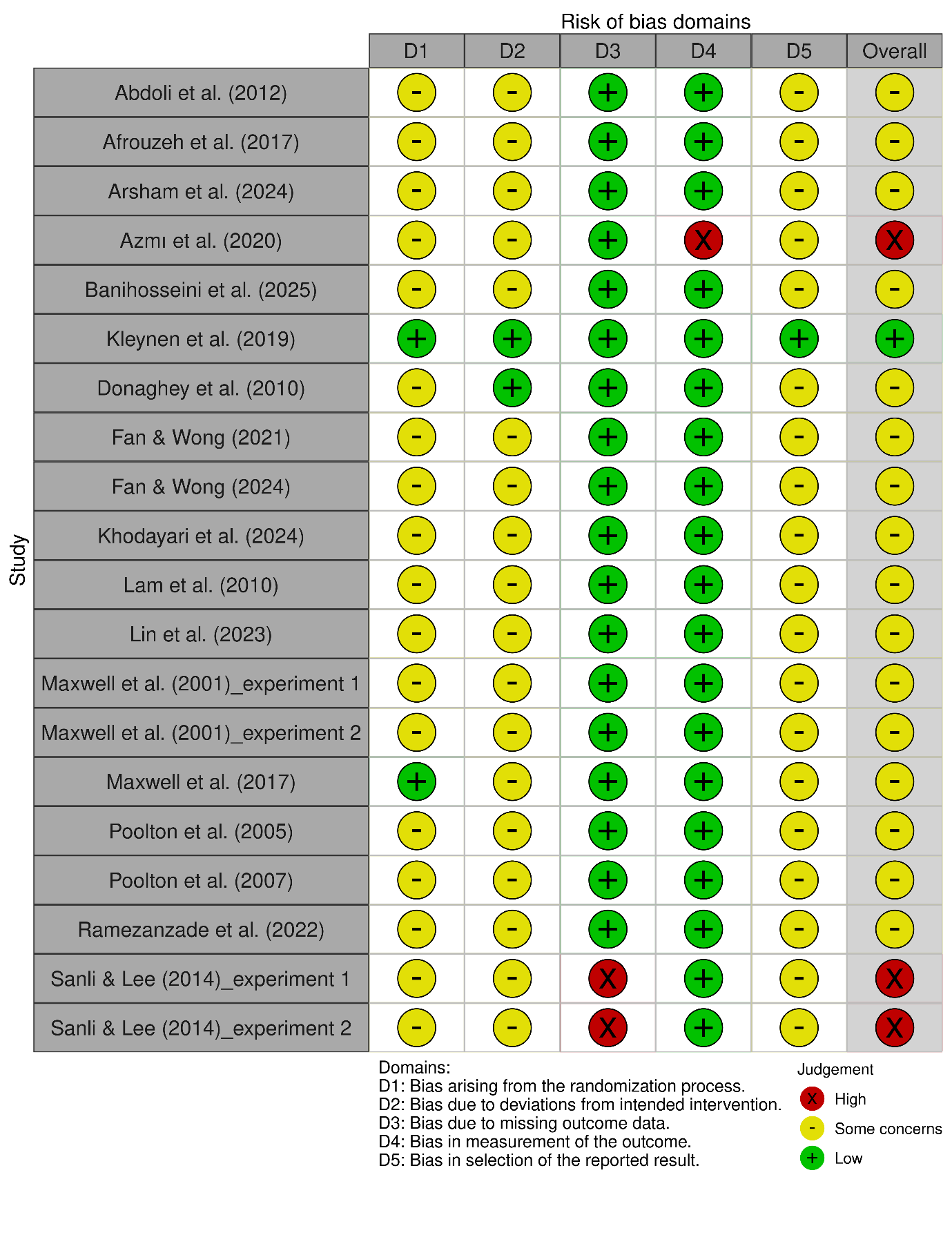


Figure S8. Risk of bias assessment of randomised parallel studies (RoB-2).


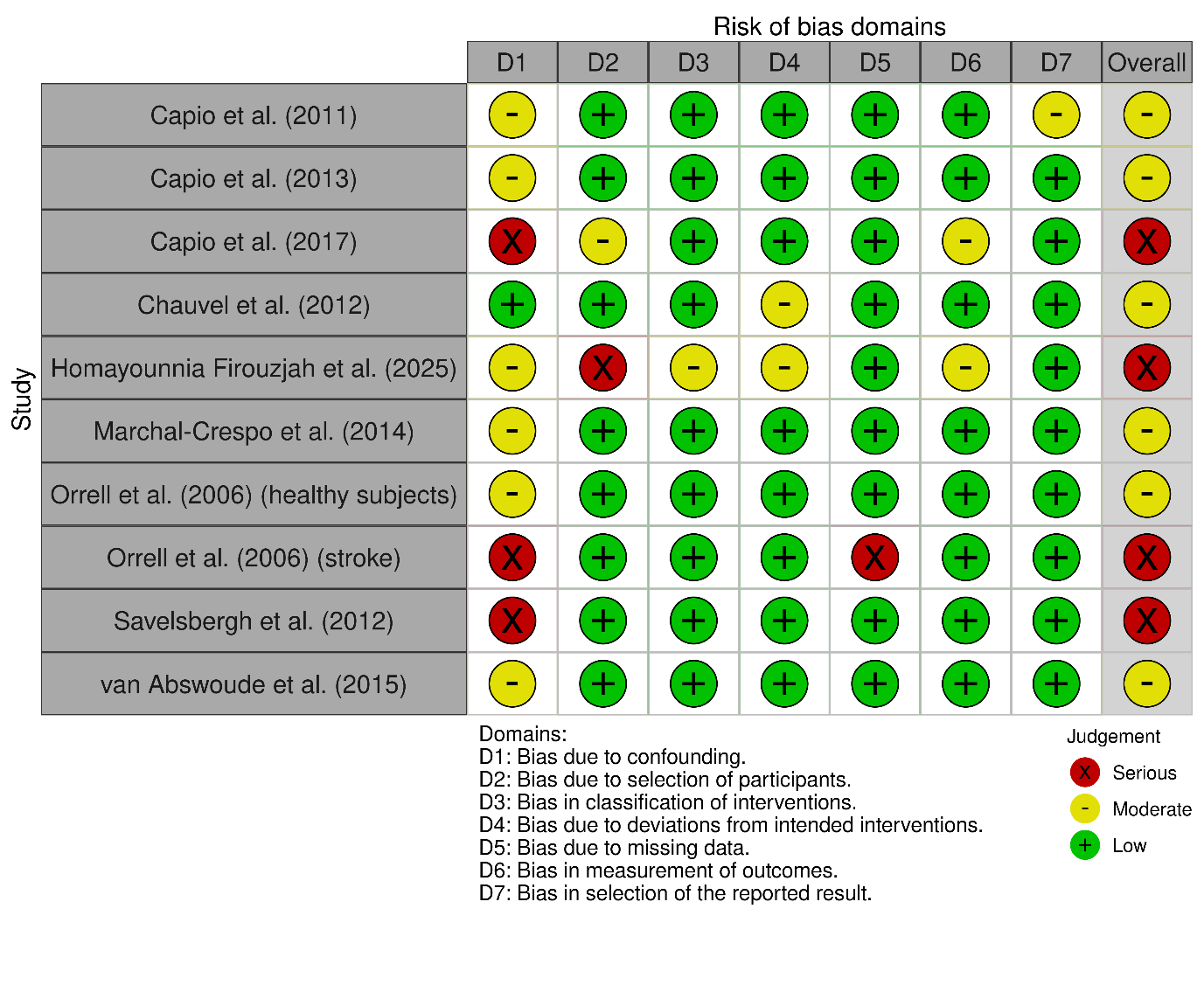


Figure S9. Risk of bias assessment of non-randomised studies (ROBINS-I V2)
