# Supplementary material for: A systematic review and meta-analysis of the effects of errorless motor learning on movement outcomes: a lifespan and impairment perspective
Source: Front Psychol. 2026 Mar 24;17:1722743. doi: 10.3389/fpsyg.2026.1722743 (PMC13053278; doi:10.3389/fpsyg.2026.1722743)
Supplement: Supplementary file 2 [file Table_2.DOCX]

| **Study** | **Setting, study design, participants** | **Task** | **Intervention** | **Outcome measures** | **Results** |
| --- | --- | --- | --- | --- | --- |
| Abdoli et al. (2012) | **Setting:** Iran  **Study design:** Randomized trial  **Total number of participants:** 30  **Inclusion/exclusion criteria:**   - No visual or motor impairment   **Number of groups:** 3   - Errorless (n=10) - Errorful (n=10) - Control (n=10)   **Demographic descriptions:**   - Gender (male/female): 0/30 - Age (years): 22 ± 2 - All participants were right-handed   **Pre-test motor performance:** N/A | Ball throwing task   - By hitting the target (shaped as concentric circle) on a board via bouncing the tennis ball off the specific target range on the ground - Scoring: ranged from 1 (outmost circle) to 10 (smallest circle) per throw | **Groups of interest:**   - Errorless: Distance from target was progressively increased (2.5-3.5m in 0.25m steps) - Errorful:   Distance from target was progressively reduced (3.5-2.5m in 0.25m steps)  **Procedure:**  *Day 1-3 (Learning phase)*   - 5 blocks 25 trials per day - End of day 3: 1 block of 10 trials at 3m distance   *Day 4 (Test phase)*   - 1 block of 10 trials at 3m - 1 block of 10 trials at 3m whilst counting backwards from 1100 in steps of 3 - 2 transfer tests (throwing from longer and shorter distance than 3m) | **Primary outcome:**  *Movement performance*   - Acquisition test: throwing precision (number of points awarded per block) (M ± SD) - Retention test: throwing precision (number of points awarded per block) (M ± SD)   **Secondary outcome:**   - Count accuracy & speed   *Verbal protocol*   - Number of explicit rules | **Movement performance:**  *Acquisition test*   - Errorless: 51.5 ± 7.706 - Errorful: 44.9 ± 9.631 - Control: 43 ± 5.206 - Errorless vs Errorful & Control: p=0.005   *Retention test*   - Errorless: 49.4 ± 4.488 - Errorful: 41.9 ± 5.32 - Control: 40.4 ± 6.84 - Errorless vs Errorful & Control: p=0.004   *Effect of group*   - η2=0.500, p=0.001 |
| Afrouzeh et al. (2017) | **Setting:** Iran  **Study design:** Randomized trial  **Total number of participants:** 40  **Inclusion/exclusion criteria:**   - No previous experiences in basketball   **Number of groups:** 2   - Errorless “ILQM” (n=20) - Explicit (n=20)   **Demographic descriptions:**   - Gender (male/female): 40/0 - Age (years): 9.93 ± 0.55 - All participants were right-handed and free of injury at the time   **Pre-test motor performance:**   - Errorless: 7.70 ± 1.78 - Explicit: 4.45 ± 1.23 | Basketball free throwing skill task   - Followed the American Alliance for Health, Physical Education, Recreation and Dance (AAPEHRD)’s basketball test - Scoring: 3 points to hit the ball into the basket without hitting the hoop or the board, 2 points to hit the ball into the basket while hitting the hoop or board, and 1 point to not hit the ball to the board or the hoop | **Groups of interest:**   - Errorless: Participants performed free throwing with a modified mini basketball (440g, 69-71cm) and rim (circumference 45 cm, height 200cm) from 300cm away from the basket - Explicit: Participants performed free throwing with a regular mini basketball (485g, 69-71cm) and rim (circumference 45cm, height 260cm) from 400cm away from the basket   **Procedure:**  *Training period*   - A total of 10 sessions - Each session contains 4 blocks of 15 trials   *Test period*   - 1 week after all trainings finished and no free throwing practice during the week | **Primary outcome:**  *Movement performance*   - Posttest: Performance score (M ± SD) - Retention test: Performance score (M ± SD)   **Secondary outcome:** N/A | **Movement Performance**  *Posttest*   - Errorless: 15.45 ± 1.19 - Explicit: 9.45 ± 1.63 - Errorless vs Explicit: t=17.031, p<0.05   *Retention test*   - Errorless: 14.75 ± 0.78 - Explicit: 8.6 ± 2.01 - Errorless vs Explicit: t=14.704, p<0.05 |
| Arsham et al. (2024) | **Setting:** Iran  **Study design:** Randomized trial  **Total number of participants:** 20  **Inclusion/exclusion criteria:**   - Diagnosis of autism from a licensed mental health clinician - Aged 9 – 13 years - No formal golf training experience - Scored higher than 70 on the WISC-IV - No significant motor impairment that would interfere with the ability to putt a golf ball, as reported by parents and confirmed by an experienced physician - No co-morbid psychiatric disorders or visual and/or auditory deficits   **Number of groups:**   - Errorless (n=10) - Explicit (n=10)   **Demographic descriptions:**   - Gender (male/female): 16/4 - Age (years): 10.15 ± 1.4 - All participants had moderate severity of autism spectrum disorder (ASD) based on the Gilliam Autism Rating Scale-2 (GARS-2)   **Pre-test motor performance:** N/A | Golf putting task | **Groups of interest:**   - Errorless: Putting distance was progressively increased (25-12 cm in 25cm steps); instructions were given at the start of the training only - Explicit: Putting distance remained at 75cm during the training; instructions were given before each block of trials     **Procedure:**  *Day 1-3 (Training phase)*   - 5 blocks, 20 trials per day   *Day 6 (Test phase)*   - 1 block, 20 trials at 75 cm - 1 block, 20 trials at 150 cm | **Primary outcome:**  *Movement accuracy*   - Performance errors, i.e. distance away from target (Mean)   *Kinematics*   - Angle of face (M ± SD) - Back-swing (M ± SD) - Top of backswing to impact (M ± SD) - Impact to the top of follow-through (M ± SD)   **Secondary outcome:** N/A | **Movement accuracy**   - Errorless: 15.11 - Explicit: 18.3 - Errorless vs Explicit: ηp2 =0.241, p=0.028   **Kinematics**  *Angle of face*   - Errorless: 5.856 ± 0.941 - Explicit: 7.014 ± 9.941   *Back-swing*   - Errorless: 9.971 ± 1.213 - Explicit: 7.090 ± 1.213   *Top of backswing to impact*   - Errorless: 4.740 ± 0.863 - Explicit: 5.999 ± 0.863   *Impact to the top of follow-through*   - Errorless: 6.338 ± 1.031 - Explicit: 7.821 ± 1.031 |
| Azmı et al. (2020) | **Setting:** Malaysia  **Study design:** Randomized trial  **Total number of participants:** 30  **Inclusion/exclusion criteria:**   - No prior experience in dart throwing   **Number of groups:**   - Errorless (n=15) - Errorful (n=15)   **Demographic descriptions:**   - Gender (male/female): 15/15 - Age (years): 21.1 ± 1.08   **Pre-test motor performance:** Yes, but no details of result were specified | Dart throwing task   - By throwing the darts to a concentric target - Scoring: ranged from 1 (outmost circle) to 3 (innermost circle), zero point for not hitting the target and the outside range of the circle | **Groups of interest:**   - Errorless: Distance from target was progressively increased (1.2-2.0m in 0.2m steps) - Errorful: Distance from target was progressively reduced (2.8-2.0m in 0.2m steps)   **Procedure:**  *Acquisition phase*   - 2 sessions with 5 blocks of 20 trials each   *Retention test*   - At least 24 hours after acquisition phase - 1 block of 20 trials at the distance of 2.0m   *Transfer test*   - 15 minutes after retention test - 1 block of 20 trials at the distance of 2.37m | **Primary outcome:**  *Movement accuracy*   - Performance consistency (M ± SD)   **Secondary outcome:** N/A | **Movement accuracy**   - Errorless: 21.9 ±1.8 - Errorful: 25.3 ± 1.8 - Errorless vs Errorful: effect size=0.422, p=0.002 |
| Banihosseini et al. (2025) | **Setting:** Iran  **Study design:** Randomized trial  **Total number of participants:** 32  **Inclusion criteria:**   - Normal vision - Right-handed - No history of psychiatric or motor disorders - Novices at the throwing task of the study   **Exclusion criteria:**   - Disagreement to continue or did not attend any of the three study sessions   **Number of groups:**   - Errorless (n=16) - Explicit (n=16)   **Demographic descriptions:**   - Gender (male/female): 12/20 - Age (years): 29 ± 5.63   **Pre-test motor performance:** No pretest was conducted | Tennis ball throwing task | **Groups of interest:**   - Errorless: Distance from target was progressively increased (2.5-3.5m in 0.25m steps) - Errorful: Distance from target was progressively reduced (3.5-2.5 m in 0.25m steps)   **Procedure:**  *Acquisition phase*   - 5 warm up trials - 5 blocks of 30 trials each   *Retention test*   - 48 hours after acquisition phase - 1 block of 10 trials at the distance of 3.0m   *Transfer test 1*   - Conducted after Stroop test - 1 block of 10 trials at the distance of 3.0m - 48 hours after retention test and transfer test 1 - 1 block of 10 trials at the distance of 3.0m after physical fatigue | **Primary outcome:**  *Movement performance*   - Throwing score (M ± SD)   **Secondary outcome:** N/A | **Movement performance**   - Errorless: 2.03 ± 0.49 - Errorful: 4.51 ± 0.65 - Effect of group: η2 = 0.300, p = 0.001, |
| Capio et al. (2011) | **Setting:** Hong Kong SAR, China  **Study design:** Quasi-experiment  **Total number of participants:** 216  **Inclusion/exclusion criteria:**   - Enrolled in PE lessons - Had not been diagnosed with any developmental conditions - No medical conditions that were contraindicated to physical activities   **Number of groups:**   - Error-reduced (n=99) - Error-strewn (n=107)   **Demographic descriptions:**   - Gender (male/female): 109/107 - Age (years): 9.16 ± 0.96   **Specific group descriptions:** N/A  **Pre-test single task motor performance:**  *Movement performance*   - Error-reduced   - High ability: 7.27 ± 0.96   - Mid-ability: 7.57 ± 1.06   - Low ability: 6.3 ± 1.59 - Error-strewn   - High ability: 7.14 ± 1.51   - Mid-ability: 7.38 ± 0.91   - Low ability: 6.53 ± 1.64   *Movement accuracy*   - Error-reduced   - High ability: 0.32 ± 0.07   - Mid-ability: 0.59 ± 0.11   - Low ability: 1.03 ± 0.29 - Error-strewn   - High ability: 0.34 ± 0.08   - Mid-ability: 0.58 ± 0.09   - Low ability: 1.01 ± 0.22 | Overhead throwing task   - By throwing beanbags to square targets of three various sizes (small, medium, and large) that were elevated from the ground at 0.8 m at 5m away from the target - Scoring: 1 point per hit into the target area | **Groups of interest:**   - Error-reduced: Size of target was progressively reduced in each session (from 2.4 x 2.4 m, 1.1 x 1.1 m, to 0.45 x 0.45 m) - Error-strewn: Size of target was progressively increased in each session (from 0.45 x 0.45 m, 1.1 x 1.1 m, to 2.4 x 2.4 m)   **Procedure:**  *Week 1 (Pretest)*   - 1 block of 10 trials   *Week 2-4 (Training phase)*   - 2 blocks of 20 trials each   *Week 5 (Test phase)*   - Posttest: 1 block of 10 trials - Transfer test: 1 block of 10 trials while counting backwards from 100 in twos | **Primary outcome:**  *Movement performance*   - Process-oriented overhead throwing scores (M ± SD) - Number of hits during practice (M ± SD)   *Movement accuracy*   - Mean absolute error from target center (M ± SD)   **Secondary outcome:** N/A | **Movement performance**  *Process-oriented overhead throwing scores*   - Error-reduced: N/A - Error-strewn: N/A   *Change in process-oriented overhand throwing scores (pretest vs posttest)*   - Error-reduced (girls): 1.67 ± 0.33 - Error-reduced (boys): 0.42 ± 0.17 - Error-strewn (girls): 0.83 ± 0.33 - Error-strewn (boys): 0.17 ± 0.21 - Changes in Error-reduced group: t=-4.51, p<0.001 - Changes in Error-strewn group: t=-2.37, p=0.02 - Effect of group: η2=0.037, p=0.05   *Number of hits during practice*   - Error-reduced (session 1): 37.67 ± 3.48 - Error-reduced (session 2): 35.17 ± 5.57 - Error-reduced (session 3): 17.00 ± 9.03 - Error-strewn (session 1): 12.32 ± 6.26 - Error-strewn (session 2): 33.20 ± 5.66 - Error-strewn (session 3): 38.05 ± 3.55   **Movement accuracy**  Change in absolute error (pretest vs posttest)   - Error-reduced (high ability): 0.03 ± 0.03 - Error-reduced (low ability): -0.38 ± 0.04 - Error-strewn (high ability): 0.14 ± 0.03 - Error-strewn (low ability): -0.25 ± 0.02 - Changes in Error-reduced group: t=2.94, p=0.005 - Changes in high ability group: t=-3.72, p<0.001 - Changes in low-ability group: t=-6.44, p<0.001 - Effect of group: η2=0.05, p=0.02 - Effect of ability: η2=0.33, p<0.001 |
| Capio et al. (2013) | **Setting:** Hong Kong SAR, China  **Study design:** Quasi-experiment  **Total number of participants:** 39  **Inclusion/exclusion criteria:**   - Diagnosis of ID - Aged between 4-11 years - Met the minimum cognitive ability requirement to be able to consistently follow twostep commands - Had normal or appropriately corrected visual acuity, and had no medical conditions that were contraindicated to physical activities   **Number of groups:**   - Error-reduced (n=18) - Error-strewn (n=21)   **Demographic descriptions:**   - Gender (male/female): 29/10 - Age (years)   - Error-reduced: 7.72 ± 2.27   - Error-strewn: 7.10 ± 1.95 - All participants had mild ID - Range of nonverbal IQs: 50-70   **Pre-test motor performance:**  *Movement form score*   - Error-reduced: 2.89 ± 0.90 - Error-strewn: 2.95 ± 2.06   *Throwing accuracy*   - Error-reduced: 0.60 ± 0.16 - Error-strewn: 0.58 ± 0.19 | Overhead throwing task   - By throwing beanbags at the center of an elevated (0.8 m above ground) cross (0.1 m long x 0.1 m wide) - Scoring: 1 point for hitting the center of the cross, none for missing it | **Groups of interest:**   - Error-reduced: Size of the target progressively reduced (1.25m x 1.25m to 0.5m x 0.5m by 0.25m x 0.25 m in each block) - Error-strewn: Size of the target rogressively increased (0.25m x 0.25m to 1.25m x 1.25m by 0.25m x 0.25m in each block)   **Procedure:**  Week 1 (Pretest)   - 1 block of 10 trials at 2.5 m from the target   Week 2-5 (Learning phase)   - Weekly session (4 in total) consisted of 3 blocks of 10 trials per session   Week 6 (Test phase)   - Posttest: 1 block of 10 trials at 2.5m from the target - Transfer test: 1 block of 10 trials at 2.5m whilst singing a familiar nursery rhyme concurrently | **Primary outcome:**  *Movement performance*   - Movement form score (M ± SD)   *Movement accuracy*   - Throwing accuracy (M ± SD)   **Secondary outcome:**   - Throwing frequency during free plays (M ± SD) | **Movement performance**   - Error-reducing: 6.00 ± 1.71 - Error-strewn: 4.67 ± 1.93 - Effect of training programme: η2=0.21, p=0.006   **Movement accuracy**   - Error-reducing: 0.47 ± 0.18 - Error-strewn: 0.51 ± 0.16 - Effect of training method: η2=0.05, p=0.20 |
| Capio et al. (2017) | **Setting:** Hong Kong SAR, China  **Study design:** Quasi-experiment  **Total number of participants:** 144  **Inclusion/exclusion criteria:**   - Children with ID had to be categorized by their school with mild ID - None specified for TD children   **Number of groups:**  TD (n=108)   - Error-reduced - Error-strewn   ID (n=36)   - Error-reduced - Error-strewn   **Demographic descriptions:**  *TD*   - Gender (male/female): 57/51 - Age (years): 9.10 ± 1.12   *ID*   - Gender (male/female): 27/9 - Age (years): 7.22 ± 2.07   **Pre-test motor performance:**  *Movement pattern score*   - *TD (Error-reduced):* 6.72 ± 1.57 - TD (Error-strewn): 6.70 ± 1.68 - ID (Error-reduced): 2.73 ± 0.80 - ID (Error-strewn): 2.78 ± 2.07   *Mastery*   - *TD (*Error-reduced*):* 47.8% - TD (Error-strewn): 46.8% - ID (Error-reduced): 0.0% - ID (Error-strewn):4.8% | Overhead throwing task   - By throwing at the target positioned at 5 m (TD group) or 2.5 m (ID group) away - Scoring: 1 point for hitting the target and 0 point for missing the target | **Groups of interest:**  TD   - Error-reduced: TD Practiced throwing at 5 m away from target that progressively decreased in size (from 2.4 × 2.4, 1.1 × 1.1, to 0.45 × 0.45 m) - Error-strewn: Practiced throwing at 5 m away from target that progressively increased in size (from 0.45 x 0.45 m, 1.1 x 1.1 m, to 2.4 × 2.4 m)   ID   - Error-reduced: Practiced throwing at 2.5 m away from target that progressively decreased in size (from 1.25 × 2.25, 1.0 × 1.0, 0.75 x 0.75 m, to 0.25 × 0.25 m) - Error-strewn: Practiced throwing at 2.5 m away from target that progressively increased in size (from 0.25 x 0.25 m, 0.75 x 0.75 m, 1.0 x 1.0 m, to 1.25 × 1.25 m)   **Procedure:**  TD   - Pretest: unspecified details - Training: 3 weekly sessions consisted of 2 blocks of 20 trials each - Posttest: unspecified details   ID   - Pretest: unspecified details - Training: 4 weekly sessions consisted of 2 blocks of 15 trials each - Posttest: unspecified details | **Primary outcome:**  *Movement performance*   - Number of successful hits during practice (M ± SD)   *Movement pattern*   - Movement pattern score (M ± SD)   *Movement stability*   - Mastery (%)   **Secondary outcome:** N/A | **Movement performance**   - *TD (*Error-reduced*):* 89.85 ± 15.75 - TD (Error-strewn): 82.41 ± 13.69 - ID (Error-reduced): 71.60 ± 22.67 - ID (Error-strewn): 51.23 ± 25.19 - Errorless vs Explicit: η2=0.10, p=0.044   **Movement pattern**   - *TD (*Error-reduced*):* 7.86 ± 0.64 - TD (Error-strewn): 7.43 ± 1.29 - ID (Error-reduced): 5.87 ± 1.81 - ID (Error-strewn): 4.44 ±1.88 - Errorless vs Explicit: Wilks’ λ = 0.91, χ2 = 8.79, p = 0.067   **Movement stability**   - *TD (*Error-reduced*):* 87.0% - TD (Error-strewn): 66.1% - ID (Error-reduced): 62.5% - ID (Error-strewn): 37.5% - Errorless vs Explicit: χ2 = 6.109, p = 0.013 |
| Chauvel et al. (2012) | **Setting:** France  **Study design:** Quasi-experiment  **Total number of participants:** 96  **Inclusion/exclusion criteria:**   - No previous golfing experience - Had normal or corrected-to-normal vision and hearing using self-report - No history of neurological disease and did not take any medication that may have affected cognition   **Number of groups:**  Young adults (n=48)   - Errorless (Infrequent) - Frequent   Older adults (n=48)   - Errorless (Infrequent) - Frequent   **Demographic descriptions:**  Young adults   - Gender (male/female): 24/24 - Age (years): 23.5 ± 3.3   Older adults   - Gender (male/female): 25/23 - Age (years): 65.0 ± 3.7   **Specific group descriptions:**   - Older adults rated themselves with poorer health than young adults - Older adults had poorer cognitive performance, and short-term and working memory than young adults   **Pre-test single task motor performance:** N/A | Golf putting task | **Groups of interest:**   - Errorless (infrequent): Distance from target was progressively increased (0.25-1.0 m in 0.25 m steps) - Frequent: Distance from target was progressively reduced (2.25-1.5 m in 0.25 m steps)   **Procedure:**  *Day 1: Learning phase*   - 4 blocks of 40 trials   *Day 1: Test phase*   - Half of errorless and frequent error groups performed a single motor transfer test - Half of the errorless (infrequent error) and frequent error groups performed a dual-task transfer test (secondary tone counting while putting) | **Primary outcome:**  *Movement performance*   - Number of successful putts in learning phase (M ± SD)   **Secondary outcome:**   - Accuracy on tone-counting task - Reports of hypothesis testing | **Movement performance**  *Number of successful putts in learning phase*   - Errorless (young adult): 29.1 ± 3.4 - Frequent (young adult): 27.0 ± 3.2 - Errorless (older adult): 27.3 ± 4.7 - Frequent (older adult): 21.3 ± 4.6 |
| Donaghey et al. (2010) | **Setting:** United Kingdom  **Study design:** Randomized controlled trial (parallel group)  **Total number of participants:** 30  **Inclusion criteria:**   - Had unilateral transtibial amputations (below the knee surgery) - Had not yet been fitted with a prosthetic limb - Had no prior knowledge of how to put on a prosthetic limb   **Exclusion criteria:**   - Neurological disorder with persisting cognitive disability, a psychiatric disorder requiring current treatment - Amputation at a level other than transtibial - Non-English-speaking participants   **Number of groups:**   - Errorless (n=15) - Control “Treatment as usual” (n=15)   **Demographic descriptions:**   - Gender (male/female): 21/9 - Age (years):   - Errorless: 62 ±14.6   - Control: 66 ± 6.8   **Specific group descriptions:**   - Most common reason for amputation was peripheral arterial disease with comorbid diabetes mellitus (n=20; 66.7%). Peripheral arterial disease (n=7; 23.3%) without comorbidity   **Pre-test motor performance:** N/A | Prosthetic limb fitting task | **Groups of interest:**   - Errorless: Sequence of prosthetic limb fitting was told to the participants to prevent the participants from making mistakes and appropriate limb-parts were given to participants to correctly move onto the next stage of fitting when required - Control: Prosthetic limb fitting was shown to participants and then they were asked to fit the limb themselves whilst errors were corrected afterwards     **Procedure:**  *Training phase*   - 5 trials   *Test phase*   - 1 videotaped trial | **Primary outcome:**  *Movement performance*   - Total number of correct steps (M ± SD)   *Movement accuracy*   - Number of omissions (M ± SD) - Number of deviations (M ± SD)   **Secondary outcome:**   - Time taken (seconds) - Number of hesitations | **Movement performance**  *Total number of correct steps*   - Errorless: 90.9 ± 12.1 - Control: 77.9 ± 8.4 - Errorless vs Control: U=28, p<0.001; d=1.25   **Movement accuracy**  *Number of omissions*   - Errorless: 0.93 ± 1.3 - Control: 2.1 ± 0.95 - Errorless vs Control: U=39, p=0.002; d=1.0   *Number of deviations*   - Errorless: 0.07 ± 0.26 - Control: 0 ± 0 - Errorless vs Control: p=0.334 |
| Fan & Wong (2021) | **Setting:** Hong Kong SAR, China  **Study design:** Randomized trial  **Total number of participants:** 36  **Inclusion/exclusion criteria:**   - Had normal or corrected to normal vision - Had no history of the retina, cerebral vascular disease, Parkinson’s disease, or any other neurological impairment before participation in the experiment   **Number of groups:**   - Errorless (n=12) - Errorful (n=12) - Control “Normal training” (n=12)   **Demographic descriptions:**   - Gender (male/female): 6/30 - Age (years): 71.06 ± 5.29   **Pre-test motor performance:**  *Movement time*   - Errorless: 9.6 ± 1.2 - Errorful: 9.6 ± 0.9 - Control: 9.6 ± 1.2   *Distance from the target*   - Errorless: 7.8 ± 0.7 - Errorful: 7.9 ± 1.1 - Control: 7.5 ± 0.5   *Jerkiness of acceleration*   - Errorless: 3.8 ± 1.7 - Errorful: 4.0 ±1.0 - Control: 4.2 ± 2.3 | Reaching motor task   - By lifting mug to reach for a specific target on a table - Scoring: parameters of movement performance and gaze behaviors were used as the outcome measures | **Groups of interest:**   - Errorless: Size of the mug handle progressively decreased (20-100 mm by 20 mm steps) - Errorful: Size of the mug handle progressively increased (100-20 mm by 20 mm steps) - Normal training: Size of the mug handle was random in each training block   **Procedure:**  *Day 1: Pretest*   - 1 block of 10 trials   *Day 1: Training phase*   - 5 blocks of 10 trials - 30-s rest intervals in between each block   *Day 1: Test phase*   - Posttest: 1 block of 10 trials with the biggest handle mug (100 mm) - Retention test 1: 1 block of 10 trials with the biggest handle mug (100 mm) after a 30-min rest following the posttest - Transfer test: 1 block of 10 trials with the biggest handle mug (100 mm) wearing special eyeglasses that simulated visual deficits - Retention test 2: 1 block of 10 trials with the biggest handle mug (100 mm) after a 60-min rest following the transfer test | **Primary outcome:**  *Movement performance*   - Movement time (s) (M ± SD)   *Movement accuracy*   - Distance away from the target   *Movement stability*   - Jerkiness of acceleration   **Secondary outcome:**  *Gaze behaviors*   - Fixation duration - Eye activity during reaching | **Movement performance**   - Errorless: 8.8 ± 1.7 - Errorful: 8.0 ± 1.5 - Control: 8.7 ± 0.3 - Errorless vs Errorful vs Control: ηp2=0.020, p=0.715   **Movement accuracy**   - Errorless: 6.0 ± 1.0 - Errorful: 7.5 ± 0.5 - Control: 7.8 ± 0.7 - Errorless vs Errorful vs Control: ηp2=0.158, p=0.059   **Movement stability**   - Errorless: 3.0 ± 0.8 - Errorful: 4.8 ± 1.0 - Control: 3.8 ± 1.9 - Errorless vs Errorful vs Control: ηp2=0.130, p=0.10 |
| Fan & Wong (2024) | **Setting:** Hong Kong SAR, China  **Study design:** Randomized trial  **Total number of participants:** 39  **Inclusion/exclusion criteria:**   - No history of the retina, cerebral vascular disease, Parkinson's disease, or any other neurological impairment   **Number of groups:**   - Errorless (n=13) - Errorful (n=13) - Normal training (n=13)   **Demographic descriptions:**   - Gender (male/female): 39/0 - Age (years): 27.03 ± 2.64   **Pre-test motor performance:**  Composite score   - Errorless: 88 ± 10 - Errorful: 90 ± 9 - Control: 87 ± 7   Jerkiness of acceleration   - Errorless: 7.5 ± 3 - Errorful: 8 ± 2.5 - Control: 9 ± 3 | Y balance lower limb reaching task   - By reaching as long as the participants could in three different reaching directions (i.e., anterior, posteromedial, and posterolateral) | **Groups of interest:**   - Errorless: Reaching distance progressively increased (20-100% of baseline distance by 20% steps) - Errorful: Reaching distance progressively decreased (100-20% of baseline distance by 20% steps) - Normal training: Reaching distance changed in a pseudorandom order in each block   **Procedure:**  *Day 1: Pretest*   - 1 block of 10 trials   *Day 1: Training*   - 5 blocks of 10 trials   *Day 1: Test phase*   - Posttest: 1 block of 10 trials - Retention test 1: 1 block of 10 trials after a 30-min rest following posttest - Transfer test: 1 block of 10 trials in untrained directions - Retention test 2: 1 block of 10 trials after a 60-min rest following transfer test | **Primary outcome:**  *Movement performance*   - Composite score (M ± SD)   *Movement stability*   - Jerkiness of acceleration (M ± SD)   **Secondary outcome:**  *Gaze behaviors*   - Fixation duration   *Balance*   - clinical test of sensory interaction and balance (CTSIB) | **Movement performance**   - Errorless: 105 ± 9 - Errorful: 94 ± 8 - Control: 88 ± 13 - Errorless vs Errorful vs Control: ηp2=0.184, p=0.036   **Movement stability**   - Errorless: 4 ± 1.5 - Errorful: 8.5 ± 1.5 - Control: 8.5 ± 3 - Errorless vs Errorful vs Control: ηp2=0.365, p<0.001 |
| Homayounnia Firouzjah et al. (2025) | **Setting:** Iran  **Study design:** Quasi-experiment  **Total number of participants:** 48  **Inclusion criteria:**   - Diagnosis of autism by a doctor based on the diagnostic and statistical guide of mental disorders - Having normal or modified vision, based on parents' reports and observing medical records available in school - No history of neurological disease or neurological disorders - IQ over 70 based on the participant's personal record as assessed by the Chinese version of the Wechsler Intelligence Scale - Fourth Edition (Wechsler, 2010) - Being right-handed - Not having any damage to torso - Having no formal training related to the skill of throwing to a circular target and no previous experience in similar tests - Being in the upper 33% range (based on cut-off point calculation) of the total scores of reinvestment scale, as the high propensity for reinvestment (high reinvestor), respectively   **Exclusion criteria:**   - Physically-motor disability - Vision and hearing defects. - Use of neuro-stimulating drugs - Being in the middle percentile of conscious reprocessing scale   **Number of groups:**   - Errorless (n=12) - Errorful (n=12) - Analogy (n=12) - Explicit (n=12)   **Demographic descriptions:**   - Gender (male/female): N/A - Age (years): 8.33 ± 0.40   **Pre-test motor performance:**  Throwing accuracy score   - Errorless: 8.1 ± 0.5 - Errorful: 7.5 ± 0.7 - Analogy: 8.3 ± 0.5 - Explicit: 7.4 ± 0.6 | Slingerball throwing task | **Groups of interest:**   - Errorless: Distance from target was progressively increased (3-5m in 1m steps) - Errorful: Distance from target was progressively decreased (5-3m in 1m steps) - Analogy: The analogy of “shoot as if you are trying to put cookies into a cookie jar on a high shelf” from distance of 4m - Explicit: Movement-related instructions were given   **Procedure:**  *Pretest*   - 20 trials at 4m   *Acquisition phase*   - 6 blocks of 10 trials   *Test phase (72-hour after acquisition phase)*   - 2 blocks of warm up - Retention test: 20 trials at 4m - Transfer test: 20 trials at 5m - Dual task test: 20 trials at 5m | **Primary outcome:**  *Movement accuracy*   - Throwing accuracy score (M ± SD)   **Secondary outcome**  *Verbal protocol*   - Number of declarative knowledge reported | **Movement accuracy**   - Errorless: 4.0 ± 1.2 - Errorful: 6.1 ± 1.7 - Analogy: 3.8 ± 1.2 - Explicit: 6.3 ± 1.5 |
| Khodayari et al. (2024) | **Setting:** Iran  **Study design:** Randomized trial  **Total number of participants:** 20  **Inclusion criteria:**   - Absence of vision and hearing impairments - No pathological conditions affecting the upper and lower limbs - Right-handed   **Exclusion criteria:**   - Lack of a signed consent form - Inability to complete the pretest - Unwillingness to participate in the exercises   **Number of groups:**   - Errorless (n=10) - Analogy (n=10)   **Demographic descriptions:**   - Gender (male/female): N/A - Age (years): 8.63 ± 2.43   **Pre-test motor performance:**  *Score*   - Errorless: 5.18 ± 2.326 - Analogy: 5.25 ± 1.832   *TGMD3 subscale test of underhand ball throwing*   - Errorless: 1.38 ± 0.744 - Analogy: 1.13 ± 0.641 | Bowling task | **Groups of interest:**   - Errorless: Distance from target was progressively increased (30-100cm in 10cm steps) - Analogy: Cartoon characters were utilized in training and training was performed from a distance of 2m   **Procedure:**  *Pretest*   - 2 trials at 3m   *Acquisition phase*   - 16 blocks of 10 trials   *Test phase*   - Retention test: 2 trials at 3m - Transfer test: 2 trials at 3m using a lighter ball | **Primary outcome:**  *Movement performance*   - Score (M ± SD)   *Movement pattern*   - TGMD3 subscale test of underhand ball throwing (M ± SD) | **Movement performance**   - Errorless: 14.13 ± 3.834 - Analogy: 13.55 ± 3.742 - Errorless vs analogy: t = 0.330, p = 0.746   **Movement pattern**   - Errorless: 2.00 ± 1.069 - Analogy: 3.53 ± 0.835 - Errorless vs analogy: z = −2.875, p = 0.004 |
| Kleynen et al. (2019) | **Setting:** Netherlands  **Study design:** Randomized controlled trial (parallel group)    **Total number of participants:** 56  **Inclusion criteria:**   - Had a stroke (>three months ago) - Capacity to walk independently with or without a walking aid over 10m (with a self-selected - Gait speed <1.2m/s) - Presence of hemiparesis (indicated by a score of <100 on the lower extremity part of the Motricity Index15 and a score <34 on the lower extremity part of the Brunnstrom Fugl-Meyer assessment - Able to visit one of the two motion capture laboratories - Had sufficient understanding of the Dutch language   **Exclusion criteria**   - Diagnosed impairments unrelated to stroke but with potential to influence gait pattern (e.g. severe osteoarthritis or amputation of the lower limb), diagnosed additional neurological impairments (e.g. Parkinson’s disease)   **Number of groups:**   - Errorless (environmental constraints) (n=17) - Analogy instructions (n=19) - Action observation (n=20)   **Demographic descriptions:**   - Gender (male/female): 32/24 - Age (years): 64.1 ± 12.0   **Pre-test motor performance:**  *Walking speed*   - Errorless: 0.542 ± 0.25 - Analogy instructions: 0.670 ± 0.23 - Action observation: 0.686 ± 0.23   *Step length (affected limb)*   - Errorless: 0.436 ± 0.08 - Analogy instructions: 0.438 ± 0.11 - Action observation: 0.443 ± 0.07   *Step length (unaffected limb)*   - Errorless: 0.328 ± 0.15 - Analogy instructions: 0.397 ± 0.15 - Action observation: 0.394 ± 0.12   *Step width (m)*   - Errorless: 0.288 ± 0.05 - Analogy instructions: 0.247 ± 0.04 - Action observation: 0.250 ± 0.06   *Step height (affected limb)*   - Errorless: 0.086 ± 0.04 - Analogy instructions: 0.098 ± 0.04 - Action observation: 0.096 ±0.04   *Step height (unaffected limb)*   - Errorless: 0.132 ± 0.02 - Analogy instructions: 0.138 ± 0.03 - Action observation: 0.134 ± 0.01   *Step length asymmetry*   - Errorless: 0.61 ± 0.11 - Analogy instructions: 0.56 ± 0.09 - Action observation: 0.57 ± 0.09   *Swing time asymmetry*   - Errorless: 0.56 ± 0.03 - Analogy instructions: 0.55 ± 0.03 - Action observation: 0.55 ± 0.03 | Walking task | **Groups of interest:**   - Errorless: Zebra crossing using black and white stripes projection on the floor and projected horizontal bar that moved at constant speed were used to control participants’ step length and walking speed respectively - Analogy instructions: participants were asked to use the 4 different analogies, which influenced walking speed, step length, step width, or step height, taught to them pictorially along with a brief introduction during the walking task - Action observation: Participants were asked to observe a short video clip that simulated the participant’s gender and ambulation method   **Procedure:**  *Pretest*   - 3 trials to establish a baseline   *Training phase*   - Errorless: 3 blocks of 3 trials - Analogy instructions: 4 blocks of 3 trials - Action observation: 3 blocks of 3 trials - Posttest: 1 trial of walking task on a 10/12 m walkway (depending on the laboratory that conducted the test) | **Primary outcome:**  *Kinematics*   - Walking speed (M ± SD) - Step length (affected limb) (M ± SD) - Step length (unaffected limb) (M ± SD) - Step height (m) (M ± SD) - Step width (affected limb) (M ± SD) - Step width (unaffected limb) (M ± SD) - Step length asymmetry (M ± SD) - Swing time asymmetry (M ± SD)   **Secondary outcome:**   - Perceived improvements in walking performance using self-developed questionnaire | **Kinematics**  *Walking speed*   - Errorless (narrow beam): 0.375 ± 0.20   - F=8.99, p=0.000 - Analogy instructions (Footprints sand; small bridge; traffic light; deep snow): 0.544 ± 0.18; 0.493 ± 0.19; 0.758 ± 0.27; 0.491 ± 0.18   - F=18.47, p=0.000: - Action observation: 0.635 ± 0.18   - F=7.95, p=0.011   *Step length (affected limb)*   - Errorless (narrow beam): 0.380 ± 0.07   - F=3.97, p=0.013 - Analogy instructions (small bridge; traffic light): 0.387 ± 0.10; 0.471 ± 0.12   - χ2 =32.42, p=0.000 - Action observation: 0.441 ± 0.06   - F=0.025, p=0.995   *Step length (unaffected limb)*   - Errorless (narrow beam): 0.274 ± 0.13   - F=5.06, p=0.004 - Analogy instructions (small bridge; traffic light): 0.352 ± 0.14; 0.436 ± 0.14   - F=12.60, p=0.000 - Action observation: 0.441 ± 0.06   - F=0.011, p=0.956   *Step width (m)*   - Errorless (narrow beam): 0.248 ± 0.06   - F=23.47, p=0.000 - Analogy instructions (footprints sand; small bridge; deep snow): 0.286 ± 0.07; 0.224 ± 0.05; 0.266 ± 0.05   - F=14.44, p=0.000 - Action observation: 0.244 ± 0.05   - F=4.70, p=0.005   *Step height (affected limb)*   - Errorless (Zebra crossing; narrow beam; moving bar): 0.082 ± 0.04; 0.072 ± 0.03; 0.079 ± 0.04   - F=6.92, p=0.003 - Analogy instructions (Traffic light; deep snow): 0.104 ± 0.04; 0.125 ± 0.8   - χ2 =29.77, p=0.000 - Action observation: 0.095 ± 0.04   - F=0.375, p=0.771   *Step height (unaffected limb)*   - Errorless (Zebra crossing; narrow beam; moving bar): 0.131 ± 0.02; 0.122 0.02; 0.127 ± 0.02   - F=2.70; p=0.080 - Analogy instructions (Small bridge; traffic light; deep snow): 0.130 ± 0.03; 0.144 ± 0.03; 0.177 ± 0.07   - F=10.65, p=0.002 - Action observation: 0.134 ± 0.01   - F=0.037, p=0.990   *Step length asymmetry*   - Errorless: p=0.024 - Analogy instructions: p=0.197 - Action observation: p=0.102   *Swing time asymmetry*   - Errorless: p=0/069 - Analogy instructions: p=0.127 - Action observation: p=0.534 |
| Lam et al. (2010) | **Setting:** Hong Kong SAR, China  **Study design:** Randomized trial  **Total number of participants:** 36  **Inclusion/exclusion criteria:**   - No previous experience in either golf putting or field hockey   **Number of groups:**   - Errorless (n=18) - Errorful (n=18)   **Demographic descriptions:**   - Gender (male/female): 22/14 - Age (years): 21.49 ± 2.03 - All participants were undergraduates from the University of Hong Kong   **Pre-test motor performance:** unspecified results | Golf putting task | **Groups of interest:**   - Errorless: Putting distance progressively increased (25-200 cm by 25 cm steps) - Errorful: Putting distance progressively decreased (200-25 cm by 25 cm steps)   **Procedure:**  *Pretest phase*   - 1 block of 20 trials without probe   *Learning phase*   - 8 blocks of 50 trials - The ball was placed approximately 20 cm away from the participants but was covered - Participants were asked to execute each putt as quickly as they wished after the ball was covered - 30 probes (auditory tone) were distributed randomly in each block of 50 trials where participants had to respond to it   *Test phase*   - Retention test 1: 1 block of 50 trials using the regular putter - Transfer test 1: 1 block of 50 trials using the “unusual putter” (88 cm long with a 4 x 2 cm cork attachment to the middle of the club face, and an 20° inclination) - Transfer test 2: 1 block of 50 trials using the “very unusual putter” (86.5 cm long with a 1.5 x 2 cm cork attachment to the middle of the club face, and a reverse 3° inclination) - Retention test 2: 1 block of 50 trials using the regular putter | **Primary outcome measures:**  *Movement performance*   - Putting score (M ± SD)   *Movement stability*   - Number of visual adjustments (M ± SD)   **Secondary outcome:**  *Reaction*   - Probe reaction time (M ± SD)   *Verbal protocol*   - Number of mechanical rules - Number of hypothesis-testing rules - Total reliance scores | **Movement performance**   - Errorless: 99.14 ± 34.56 - Errorful: 84.19 ± 33.42 - Errorless vs Errorful: η2=0.05, p=0.20   **Movement stability**   - Errorless: 3.44 ± 3.15 - Errorful: 2.67 ± 2.52 |
| Lin et al. (2023) | **Setting:** Taiwan  **Study design:** Randomized trial  **Total number of participants:** 28  **Inclusion/exclusion criteria:**   - No history of neuromuscular diseases   **Number of groups:**   - Error-reduction (n=14) - Control (n=14)   **Demographic descriptions:**   - Gender (male/female): 15/17 - Age (years): 24.6 ± 1.6 - All participants were right-handed   **Pre-test motor performance:**  *Task accuracy* (*root mean squared of task error)*   - Error-reduction: 0.992 ± 0.247 - Control: 0.942 ± 0.307   *Task accuracy* *(combined amplitudes of 0.2 Hz and 0.5 Hz spectral peaks in the force output)*   - Error-reduction: 0.814 ± 0.171 - Control: 0.819 ± 0.273 | Visuo-motor task (bi-rhythmic task)   - By exerting isometric index abduction of dominant hand using combined sinusoidal forces of 0.2 Hz and 0.5 Hz that fluctuated around 30% ± 2% MVC | **Groups of interest:**   - Error-reduction: Participants received feedback that was reduced to half the size of the no reduction in errors during training where the visualized force on screen was the sum of half of the real force (RF) plus half of the target signal (T) - Control: Participants were trained with visual feedback that guided force-tracking with feedback with no reduction in errors   Procedure:  Day 1: Pretest   - 3 trials of 3-s isometric index abduction to determine participant’s maximal voluntary contraction (MVC) - 3 trials of bi-rhythmic force task under visual feedback   Day 2: Training phase   - 15 trials of bi-rhythmic task   Day 3: Test phase   - 3 trials of bi-rhythmic task | **Primary outcome:**  *Movement accuracy*   - Task accuracy (root mean squared of task error) (M ± SD) - Task accuracy (combined amplitudes of 0.2 Hz and 0.5 Hz spectral peaks in the force output) (M ± SD)   **Secondary outcome:**  *EMG*   - Variables of inter-spike interval | **Movement accuracy (root mean squared of task error)**   - Error-reduction: 0.910 ± 0.265 - Control: 0.690 ± 0.176   **Movement accuracy (combined amplitudes of 0.2 Hz and 0.5 Hz spectral peaks in the force output)**   - Error-reduction: 0.837 ± 0.241 - Control: 1.041 ± 0.215 |
| Marchal-Crespo et al. (2014) | **Setting:** Switzerland  **Study design:** Within-group, crossover design  **Total number of participants:** 22    **Inclusion/exclusion criteria:** N/A  **Number of groups (crossover study):**   - Errorless (hepatic guidance) (n=22) - No guidance (n=22) - Error-amplification (n=22) - Noise disturbance (n=22)   **Demographic descriptions:**   - Gender (male/female): 22/0 - Age (years): 23.0 ± 2.0 - All participants were right-footed   **Pre-test motor performance:** N/A | Locomotor task   - By performing gait-like stepping movements on a foot plate whilst attached to the fMRI compatible robotic stepping actuator | **Groups of interest:**   - Hepatic guidance: Position controller enforced the desired knee trajectory via the length of the knee cylinder - No guidance: The robot was compliant to the participant’s self-initiated movements - Error-amplification: The robot amplified the errors generated when trying to follow the desirable knee movement and amount of force given would be proportional to the size of tracking error - Noise disturbance: Random force was applied to the knee to evoke errors   **Procedure:**  *Learning phase*   - 30 trials of 9-s of movement   *Test phase*   - 100-s of sustained movement - Retention test was completed right after completion of the learning phase of each group and then followed by the other learning phase of another group | **Primary outcome:**  *Movement accuracy*   - Mean tracking error   *Muscle efficiency*   - Muscle activation (% MVC)   **Secondary outcome:** N/A | **Movement accuracy**   - Errorless (skilled/non-skilled): 0.027 ± 0.004 / 0.04 ± 0.004 - No guidance (skilled/non-skilled): 0.028 ± 0.006 / 0.034 ± 0.002 - Error-amplification (skilled/non-skilled): 0.029 ± 0.005 / 0.0295 ± 0.0045 - Noise disturbance (skilled/non-skilled): 0.024 ± 0.001 / 0.032 ± 0.004   **Muscle efficiency**   - Errorless: 7.55% - No guidance: 7.75% - Error-amplification: 8.75% - Noise disturbance: 8.55% |
| Maxwell et al. (2001) _***experiment 1*** | **Setting:** United Kingdom  **Study design:** Randomized trial  **Total number of participants:** 29  **Inclusion/exclusion criteria:**   - No previous golfing experience   **Number of groups:**   - Errorless (n=11) - Errorful (n=9) - Random (n=9)   **Demographic descriptions:**   - Gender (male/female): N/A - Age (years): 20.9±2.4   **Pre-test motor performance:** N/A | Golf-putting task | **Groups of interest:**   - Errorless: Distance from target was progressively increased (25-200 cm in 25 cm steps) - Errorful: Distance from target was progressively reduced (200-25 cm in 25 cm steps) - Random: Similar to that of errorful group in a qualitative way   **Procedure:**  *Day 1: Learning phase*   - 8 blocks of 50 trials   *Day 1: Test phase*   - Retention test: 1 block of 50 trials at 200 cm - Transfer test 1: 1 block of 50 trials at 200 cm while monitoring and subsequently reported the number of high-pitched tones present among high and low-pitched tones - Transfer test 2: 1 block of 50 trials at 300 cm | **Primary outcome:**  *Movement performance*   - Number of successful putts (M ± SD)   **Secondary outcome:**  *Accuracy*   - Tone counting accuracy (%)   *Verbal protocol*   - Number of explicit rules (M ± SD) | **Movement performance**   - Errorless: 41.36 ± 6.61 - Errorful: 36.56 ± 4.75 - Control: 34.56 ± 4.90 - Errorless vs Errorful & Control: t=2.721, p=0.01 |
| Maxwell et al. (2001) _***experiment 2*** | **Setting:** United Kingdom  **Study design:** Randomized trial  **Total number of participants:** 55  **Inclusion/exclusion criteria:**   - No previous golfing experience   **Number of groups:**   - Errorless experimental (n=14) - Errorless control (n=13) - Errorful experimental (n=14) - Errorful control (n=14)   **Demographic descriptions:**   - Gender (male/female): N/A - Age (years): 21.0 ± 2.8   **Pre-test motor performance:** N/A | Golf putting task | **Groups of interest:**   - Errorless: Distance from target was progressively increased (25-75 cm in 25 cm steps) - Errorful: Distance from target was progressively reduced (175-125 cm in 25 m steps)   **Procedure:**  Day 1: Learning phase   - 3 blocks of 50 trials   Day 1: Test phase   - Transfer test (control groups): 1 block of 50 trials at 100 cm - Transfer test (experimental group): 1 block of 50 trials while monitoring and subsequently reported the number of high-pitched tones present among high and low-pitched tones | **Primary outcome:**  *Movement performance*   - Number of successful putts (M ± SD)   *Movement accuracy*   - Number of errors in learning phase (M ± SD)   **Secondary outcome:**  *Accuracy*   - Tone counting accuracy (%)   *Verbal protocol*   - Number of explicit rules (M ± SD) | **Movement performance**   - Errorless: 40 - Errorful: 25   **Movement accuracy**   - Errorless (experimental): 18 - Errorless (control): 10 - Errorful (experimental): 90 - Errorful (control): 96 |
| Maxwell et al. (2017) | **Setting:** Hong Kong SAR, China  **Study design:** Randomized trial  **Total number of participants:** 45  **Inclusion/exclusion criteria:**   - No previous experience in playing golf   **Number of groups:**   - Errorless High-Ability (n=13) - Errorless Low-Ability (n=11) - Errorful High-Ability (n=10) - Errorful Low-Ability (n=11)   **Demographic descriptions:**   - Gender (male/female): 25/20 - Age (years): 9.80 ± 0.59   **Pre-test motor performance:** N/A | Golf putting task   - Scoring: 3 points = in the hole; 2 points = within 13 cm of the hole; 1 point = within 26 cm of the hole; 0 point = all other trials | **Groups of interest:**   - Errorless: Distance from target was progressively increased (25-150 cm in 25 cm steps) - Errorful: Distance from target was progressively reduced (150-25 cm in 25 m steps)   **Procedure:**  Day 1: Learning phase   - 6 blocks of 50 trials - Day 1: Test phase - Retention test 1: 1 block of 50 trials at 150 cm - Transfer test: 1 block of 50 trials at 150 cm while counting high-pitch tones they had heard - Retention test 2: 1 block of 50 trials at 150 cm | **Primary outcome:**  *Movement performance*   - Golf putting scores (M ± SD)   **Secondary outcome:**  *Accuracy*   - Tone-counting accuracy (M ± SD)   *Verbal protocol*   - Number of explicit rules (M ± SD) | **Movement performance**   - Errorless High-Ability: 50 ± 5 - Errorless Low-Ability: 49 ± 5 - Errorful High-Ability: 58 ± 4 - Errorful Low-Ability: 43 ± 5 |
| Mount et al. (2007) | **Setting:** United States of America  **Study design:** Randomized controlled trial (crossover)  **Total number of participants:** 33  **Inclusion criteria:**   - diagnosis of acute stroke confirmed by computerized tomography, magnetic resonance imaging, or clinical examination.   **Exclusion criteria:**   - Demonstrated proper technique for wheelchair set up without instruction or if they were previously taught the sock-donning task upon initial evaluation - No physical or perceptual ability to complete either task with verbal instructions due to severe weakness, unilateral neglect, apraxia, or spatial deficits - Documented history of aphasia - Inability to understand instructions in English - Obesity or co-morbidities that prevented sock-donning task to be attempted - Had an expected length of stay in the hospital (i.e. which was less than one week from the time of screening)   **Number of groups:**   - Errorless wheelchair (n=16) - Errorless sock-donning (n=17) - Trial-and-error wheelchair (n=16) - Trial-and-error sock-donning (n=17)   **Demographic descriptions:**   - Gender (male/female): 18/15 - Age (years): 63 ± 12   **Pre-test motor performance:** N/A | - Wheelchair preparation task - Sock-donning task | **Groups of interest:**   - Errorless: Participants were told to perform each step only if they were confident and instructors would provide hand-on-hand instruction with verbal cues when the participants were not sure - Trial-and-error: Participants were allowed to make mistakes during the task sequence and verbal cues were provided to correct the errors   **Procedure:**  Day 1-7: Learning phase   - 2 trials per day - Discontinued if retention was not achieved after day 7   Day 1-8: Test phase   - Retention test (day 1-7): performed at the end of each training day - Carry-over task (1 day after retention was achieved): 1 trial of using a different type of wheelchair or sock donner | **Primary outcome:**  *Movement performance*   - Incidence rate of retention   **Secondary outcome:**  *Errors in learning*   - Number of verbal errors - Number of errors of sequence Number of errors of action - Number of errors of no response | **Movement performance**  *Wheelchair task*   - Errorless (impaired memory): 0.46 - Errorless (intact memory): 0.25 - Trial-and-error (impaired memory): 0.21 - Trial-and-error (intact memory): 0.44   *Sock-donning task*   - Errorless (impaired memory): 0.26 - Errorless (intact memory): 0.32 - Trial-and-error (impaired memory): 0.33 - Trial-and-error (intact memory): 0.27 |
| Orrell et al. (2006) – ***healthy subjects*** | **Setting:** United Kingdom  **Study design:** Quasi-experiment  **Total number of participants:** 42 (6 dropouts)  **Inclusion/exclusion criteria:**   - No previous experience pf surfing, snowboarding or other similar balancing tasks   **Number of groups:**   - Errorless (n=13) - Discovery learning (n=12) - Analogy (n=11)   **Demographic descriptions:**   - Gender (male/female): 17/19 - Age (years): 20.29 ± 1.17   **Pre-test motor performance:** N/A | Balancing task   - Keeping a 1- axial stabilometer platform horizontal for 60-s | **Groups of interest:**   - Errorless: no further instruction other than keeping the stabilometer platform horizontal was given to participants - Discovery learning: Participants were instructed to discover the rules of how to perform the task - Analogy: Participants were instructed using analogy (i.e. to pretend they were soldiers standing on guard outside Buckingham Palace)   **Procedure:**  *Day 1: Learning phase*   - 16 60-s trials, with 1-min rest interval between each trial   *Day 1-2: Test phase*   - Retention test: 2 60-s trials alone - Transfer test 1: 1 60-s trial while recalling random 7-digit sequences presented at a rate of 1 digit sequence per second at the last 30-s of the trial - Transfer test 2: 1 60-s trial while reaching out to pick up and hold a 2 kg kettle at the last 30-s of the trial - Delayed retention test (day 2): 2 60-s trials alone - Transfer test 3: 1 60-s trial while performing a concurrent tone counting task throughout the 60-s and picking up and holding a 2 kg kettle with 1 hand at the last 30-s of the trial | **Primary outcome measures:**  *Movement accuracy*   - Mean square error (RMSE) in degrees   **Secondary outcome:**  *Verbal protocol*   - Number of explicit rules | **Movement accuracy**   - Errorless: 3.25 - Discovery learning: 2.75 - Analogy: 3.35 - Effect of group: Wilk’s Λ = 0.968, p = 0.58 |
| Orrell et al. (2006) – ***stroke patients*** | **Setting:** United Kingdom  **Study design:** Quasi-experiment  **Total number of participants:** 22  **Inclusion/exclusion criteria (stroke group):**   - Diagnosis of first stroke at least 12 months prior to study to reduce potential spontaneous recovery confounding the data - Discharged from all rehabilitation services - Ability to understand instructions and to give informed consent - No obvious cognitive or perceptual problems on the MiniMental State Examination   **Number of groups:**   - Errorless stroke (n=5) - Discovery learning stroke (n=5) - Errorless control (n=6) - Discovery learning control (n=6)   Demographic descriptions:   - Gender (male/female): 15/7 - Age (years):   - Stroke: 52.17 ± 11.27   - Control: 65.25 ± 7.48   **Pre-test motor performance:** N/A | Dynamic balancing task   - Keeping a 1- axial stabilometer platform horizontal for 60-s | **Groups of interest:**   - Errorless: A 2.5 kg braking resistance was applied to restrict full movement of the stabilometer platform and resistance gradually reduced by 0.5 kg every 4 trial until no resistance at the final 4 trials - Discovery learning: Participants were instructed to discover the rules of how to perform the task   **Procedure:**  *Day 1: Learning phase*   - 24 60-s trials, with 1-min rest interval between each trial   *Day 1-2: Test phase*   - Retention test 1: 2 60-s trials alone - Transfer test 1: 1 60-s trial while recalling random 6-digit sequences presented at a rate of 1 digit sequence per second at the last 30-s of the trial - Retention test 2: 2 60-s trials alone - Transfer test 2: 1 60-s trial while reaching out to pick up and hold a 1 kg kettle at the last 30-s of the trial - Delayed retention test (day 2): 2 60-s trials alone | **Primary outcome measures:**  *Movement accuracy*   - Mean square error (RMSE) in degrees   **Secondary outcome:**  *Verbal protocol*   - Number of explicit rules | **Movement accuracy**  *Retention test 1*   - Errorless stroke: 7.5 - Discovery stroke: 8.5 - Errorless control: 6.5 - Discovery control: 7.0   *Retention test 3*   - Errorless stroke: 8.8 - Discovery stroke: 9.0 - Errorless control: 5.9 - Discovery control: 6.0   *Effect of group x condition x block*   - F=2.39, P=0.10, df=2,17 |
| Poolton et al. (2005) | **Setting:** Hong Kong SAR, China  **Study design:** Randomized trial  **Total number of participants:** 35  **Inclusion/exclusion criteria:**   - No previous experience in golfing   **Number of groups:**   - Errorless “Implicit-Explicit” (n=17) - Explicit (n=18)   **Demographic descriptions:**   - Gender (male/female): 11/24 - Age (years): 21.1 ± 1.48   **Pre-test motor performance:** N/A | Golf putting task | **Groups of interest:**   - Errorless: Distance was progressively increased (25-200 cm by 25 cm steps) and verbal instructions were given to participants prior to 4^th^ block of learning phase - Explicit: Distance was progressively increased (25-200 cm by 25 cm steps) and verbal instructions were given to participants before learning   **Procedure:**  *Day 1: Learning phase*   - 8 blocks of 50 trials   *Day 1: Test phase*   - Retention test 1: 1 block of 50 trials at 200 cm - Transfer test: 1 block of 50 trials at 200 cm while performing a tone-counting task - Retention test 2: 1 block of 50 trials at 200 cm | **Primary outcome:**  *Movement performance*   - Number of successful putts   *Movement stability*   - Number of techniques change   **Secondary outcome:**  *Verbal protocol*   - Number of explicit rules | **Movement performance**  *Retention test 1*   - Errorless: 28.23 - Explicit: 29.98   *Retention test 2*   - Errorless: 27.5 - Explicit: 30.0   *Effect of group*   - η2=0.002, p=0.79   **Movement stability**  *Retention test 1*   - Errorless: 1.5 - Explicit: 3.4   *Retention test 2*   - Errorless: 1 - Explicit: 2.8 |
| Poolton et al. (2007) | **Setting:** Hong Kong SAR, China  **Study design:** Randomized trial  **Total number of participants:** 56 (1 person excluded in the errorful condition)  **Inclusion/exclusion criteria:**   - No previous experience with rugby passing task   **Number of groups:**   - Errorless (n=23) - Errorful (n=22) - Control (n=10)   **Demographic descriptions:**   - Gender (male/female): 23/22 - Age (years): 23 ± 4.95   **Pre-test motor performance:** N/A | Rugby passing task   - By throwing rugby ball underhand at an elevated target (125 cm tall) consisted of 3 concentric squares (30, 100, & 150 cm) | **Groups of interest:**   - Errorless: Distance was progressively increased (1.0-3.0 m by 0.5 m steps) - Errorful: Distance was progressively decreased (6.0-4.0 m by 0.5 m steps)   **Procedure:**  *Day 1: Learning phase*   - 10 blocks of 10 trials   *Day 1: Test phase*   - Retention test 1: 1 block of 10 trials at 3.5 m - Transfer test: 1 block of 10 trials at 3.5 m while performing a letter generation task concurrently - Retention test 2: 1 block of 10 trials at 3.5 m - Fatigue-performance test: 2 trials of Wingate Anaerobic Tests (WAnT) | **Primary outcome:**  *Movement accuracy*   - Distance from target in mm (M)   **Secondary outcome:**  *Verbal protocol*   - Number of explicit rules | **Movement accuracy**   - Errorless: 213.3 - Errorful: 189.7 - Effect of group: η2=0.002, p=0.76 |
| Ramezanzade et al. (2022) | **Setting:** Iran  **Study design:** Randomized trial  **Total number of participants:** 120  **Inclusion/exclusion criteria:**   - Right-handed - Healthy - Normal eyesight or wore modified glasses - No previous experience in dart throwing   **Number of groups:**   - Random practice (n=15) - Random Errorless (n=15) - Random Errorful (n=15) - Serial Errorless (n=15) - Serial Errorful (n=15) - Block Errorless (n=15) - Block Errorful (n=15) - Constant practice (n=15)   **Demographic description:**   - Gender (male/female): 120/0 - Age (years): 21.19 ± 1.4   **Pretest motor performance:** Random practice: 5.0 ± 0.1  Random-Errorless: 5.0 ± 0.2  Random-Errorful: 5.2 ± 0.3  Serial-Errorless: 5.0 ±0.2  Serial-Errorful:4.9 ± 0.4  Block-Errorless: 5.2 ± 0.3  Block-Errorful: 5.0 ± 0.4  Constant practice: 5.1 ± 0.3 | Dart throwing task | **Groups of interest:**   - Random practice: throwing distance was randomly given between 137-337 cm - Random-Errorless: random throwing distance but distance was from close to far from target (chosen between 137-337 cm) - Random Errorful: random throwing distance and distance gradually moved from far from target to close to target (chosen between 137-337 cm) - Serial Errorless: Throwing distance gradually increased in each trial (137-337 cm by 33.33 cm steps) and repeated in later trials - Serial Errorful: Throwing distance gradually decreased in each trial (337-137 cm by 33.33 cm steps) and repeated in later trials - Block Errorless: Throwing distance was progressively increased in each block (137-337 cm by 33.33 cm steps) - Block Errorful: Distance was progressively decreased (337- 137 cm by 33.33 cm steps) - Constant practice: Constant throwing distance throughout   **Procedure:**  *Day 1-3: Acquisition phase*   - Pretest (10 throws from 237 cm) - 210 trials across 3 days (70 trials per each training day)   *Day 3: Testing phase 1*   - Acquisition test: 10 throws from 237 cm - Transfer test 1: 10 throws at 237 cm with a secondary task   *48 hours after Day 3: Testing phase 2*   - Transfer test 2: 10 throws at 237 cm - Retention test: 10 throws at 237 cm | **Primary outcome:**  *Movement accuracy*   - Performance error (i.e. distance from target) (M ± SD) | **Movement accuracy**   - Random practice: 2.6 ± 0.2 - Random-Errorless: 1.8 ± 0.3 - Random-Errorful: 4.5 ± 0.3 - Serial-Errorless: 3.9 ± 0.3 - Serial-Errorful: 3.0 ± 0.3 - Block-Errorless: 4.0 ± 0.2 - Block-Errorful: 2.8 ± 0.3 - Constant practice: 4.2 ± 0.2 - Effect of group: ηp2=0.662, P=0.001, sum of squares=87.032, mean square=12.433, 95% CI=3.133-3.523 |
| Sanli & Lee (2014) -***experiment 1*** | **Setting:** Canada  **Study design:** Randomized trial  **Total number of participants:** 19  **Inclusion/exclusion criteria:**   - Not specified   **Number of groups:**   - Errorless “large-to-small target” (n=10) - Errorful “small-to-large target” (n=9)   **Demographic descriptions:**   - Gender (male/female): 12/9 - Age (years): 25.6 ± 3.2   **Pre-test motor performance:** N/A | Aiming task   - By propelling a small disc over a smooth tabletop while aiming to stop it within a specified target circle | **Groups of interest:**   - Errorless: Target size progressively reduced (31-6.5 cm diameter, in 3.5 cm steps) - Errorful: Target size progressively increased (6.5-31 cm diameter, in 3.5 cm steps)   **Procedure:**  *Day 1: Acquisition phase*   - 200 acquisition trials (25 to each of 8 target sizes)   *Day 1: Test phase 1*   - Retention test: 25 trials at the 6.5 cm target size - Immediate dual-task test: 25 trials at the 6.5 cm target size while performing tone-counting task concurrently - Immediate transfer test: 25 trials at the 4.5 cm target size   *Day 2: Test phase 2*   - Identical to test phase 1 | **Primary outcome:**  *Movement accuracy*   - Proportion of errors (i.e., number of times the disc did not stop completely within the target area) (M± SD)   **Secondary outcome:**   - Tone counting accuracy | **Movement accuracy**  *Test phase 1*   - Errorless: 0.81 - Errorful: 0.82   *Test phase 2*   - Errorless: 0.83 - Errorful: 0.81   *Errorless vs Errorful (Test phase 1 and 2 combined)*   - p>0.05 |
| Sanli & Lee (2014) - ***experiment 2*** | **Setting:** Canada  **Study design:** Randomized trial  **Total number of participants:** 20  **Inclusion/exclusion criteria:**   - Not specified   **Number of groups:**   - Errorless “near-to-far" (n=10) - Errorful “far-to-near” (n=10)   **Demographic descriptions:**   - Gender (male/female): 8/12 - Age (years): 21.2 ± 2.9   **Pre-test motor performance:** N/A | Aiming task   - Subjects needed to propel small disc over a smooth tabletop, while aiming to stop it in a specified target circle of 6.5 cm in diameter | Groups of interest:   - Errorless: Progressively increased distance from target (from 3.5, 7.5, 11.5, 15.5, 18.5, 22.5, 26.5, and 30.5 cm distance) - Errorful: Progressively reduced distance from target (from 30.5, 26.5, 22.5, 18.5, 15.5, 11.5, 7.5, and 3.5 cm distance)   Procedure:  **Groups of interest:**   - Errorless: Target size progressively reduced (31-6.5 cm diameter, in 3.5 cm steps) - Errorful: Target size progressively increased (6.5-31 cm diameter, in 3.5 cm steps)   **Procedure:**  *Day 1: Acquisition phase*   - 200 acquisition trials (25 to each of 8 target sizes)   *Day 1: Test phase 1*   - Retention test: 25 trials at the 6.5 cm target size - Immediate dual-task test: 25 trials at the 6.5 cm target size while performing tone-counting task concurrently - Immediate transfer test: 25 trials at the 4.5 cm target size   *Day 2: Test phase 2*   - Identical to test phase 1 | **Primary outcome:**  *Movement accuracy*   - Proportion of errors (i.e., number of times the disc did not stop completely within the target area) (M± SD)   **Secondary outcome:**   - Tone counting accuracy | **Movement accuracy**  *Test phase 1*   - Errorless: 0.89 - Errorful: 0.88   *Test phase 2*   - Errorless: 0.95 - Errorful: 0.87   *Errorless vs Errorful (Test phase 1 and 2 combined)*   - p>0.05 |
| Savelsbergh et al. (2012) | **Setting:** Netherlands  **Study design:** Quasi-experiment  **Total number of participants:** 40  **Inclusion/exclusion criteria:**   - Able to kick the ball from the midline of the soccer field across the goal line (i.e., a distance of 50 m)   **Number of groups:**   - Errorless “Increasing difficulty” (n=10) - Random difficulty (n=10) - Constant difficulty (n=10) - Decreasing difficulty (n=10)   **Demographic descriptions:**   - Gender (male/female): 40/0 - Age (years): 20.5 ± 0.7   **Pre-test motor performance:**   - Errorless: 72 - Random difficulty: 76 - Constant difficulty: 71 - Decreasing difficulty: 62 | Soccer free-kicking task   - 4 free-kick conditions of increasing difficulty for practice  1. A low shot at the target area from a distance of 9 m 2. Curling (i.e., shooting a high ball) the ball from a distance of 9 m 3. Curling the ball from a distance of 12.3 m 4. Curling the ball from a distance of 15.6 m 5. curling the ball from a distance of 18.9 m  - Scoring: 5 points = the ball hit the specific target area; 3 points = the ball nearly hits the target area, but would have been out of reach of the goalkeeper; 1 point = the ball clearly missed the target area, but hit the goalmouth at a location where it is within reach of the goalkeeper ; 0 points = the ball completely missed the goal | **Groups of interest:**   - Errorless: Participants performed the task in increasing difficulty - Random difficulty: Participants performed the task in random difficulty - Constant difficulty: Participants performed the task in only the most difficult condition - Decreasing difficulty: Participants performed the task in decreasing difficulty   **Procedure:**  *Learning phase*   - 5 blocks of 15 trials   *Test phase*   - Test 1: 1 block of 15 trials with 2-men practice wall present - Test 2: 1 block of 15 trials with 5-men practice wall present | **Primary outcome:**  *Movement performance*   - Number of free-kick points   **Secondary outcome:** N/A | **Movement performance**  *Posttest*   - Errorless: 97 - Random difficulty: 70 - Constant difficulty: 80 - Decreasing difficulty: 66   *Retention test*   - Errorless: 90 - Random difficulty: 69 - Constant difficulty: 88 - Decreasing difficulty: 69   *Effect of group x test*   - η2=0.12, p<0.05 |
| van Abswoude et al. (2015) | **Setting:** Netherlands  **Study design:** Quasi-experiment  **Total number of participants:** 38  **Inclusion/exclusion criteria:**   - Diagnosis of cerebral palsy confirmed by either the physical therapist or the physical education teacher - Ale to throw a ball (however accurate) - Able to understand the instructions of the experimenter. - Able to verbally respond to questions   **Number of groups:**   - Error-minimizing (n=20) - Error-strewn (n=18)     **Demographic descriptions:**   - Gender (male/female): 18/20 - Age (years): 9.5 ± 4.0   **Pre-test motor performance:** N/A | Aiming task   - By rolling plastic ball through the space between 2 foam block aperture placed 3 m in front of the participant | **Groups of interest:**   - Error-minimizing: Distance between the aperture progressively decreased (90-30 cm by 20 cm steps) - Error-strewn: Distance between the aperture progressively increased (30-90 cm by 20 cm steps)   **Procedure:**  Day 2: Learning phase   - 4 blocks of 20 trials - Day 2: Test phase - Posttest: 1 block of 10 trials with aperture width of 15 cm - Retention test: 1 block of 10 trials with no aperture - Transfer test: 1 block of 10 trials with aperture width of 15 cm while performing counting task | **Primary outcome:**  *Movement accuracy*   - Number of errors during practice (M ± SD)   **Secondary outcome:** N/A | **Movement accuracy**   - Error-minimizing: 30.50 ± 13.80 - Error-strewn: 25.44 ± 12.47 |

*ILQM* Implicit learning of equipment modification, *WISC-IV* Wechsler Intelligence Scale for Children-Fourth Edition, *ID* Intellectual disability, *TD* Typically developed
